# Light-Assisted Conversion of Aqueous CO_2_ (Bicarbonate) into Surface Reduced Species by a CeO_2_–SnO_2_ Hybrid

**DOI:** 10.1021/acsomega.6c00620

**Published:** 2026-04-27

**Authors:** Lucas Hansen, Ubiratan Hack, Marco Antônio Rodrigues Siqueira, Daniel Eduardo Weibel

**Affiliations:** † Instituto de Química, 28124 Universidade Federal do Rio Grande do Sul, Avenida Bento Gonçalves 9500, Porto Alegre, RS 91501-970, Brazil; ‡ Laboratório Aquário, 125098 Universidade Feevale, RS 239 2755, Novo Hamburgo, RS 93525-075, Brazil

## Abstract

Understanding the interfacial chemistry of bicarbonate at oxide surfaces is central to advancing photodriven carbon transformation processes. Here, a CeO_2_–SnO_2_ hybrid oxide synthesized via a simple solid-state route is investigated as a model system for UV–visible-light-assisted bicarbonate conversion at a fundamental, proof-of-concept level. Surface-sensitive ATR-FTIR spectroscopy reveals a progressive enhancement of formate-related vibrational features under UV–visible-light irradiation of the hybrid material, whereas dark-treated samples and physical mixtures of the parent oxides remain dominated by overlapping carbonate- and water-related absorptions. Bayesian inference applied to overlap-corrected FTIR band areas, independently corroborated by unsupervised self-organizing map analysis of the full spectral fingerprints, indicates that the sustained accumulation of formate-like species is intrinsic to the hybrid surface chemistry rather than inherited from the individual oxides. Total organic carbon measurements show no measurable accumulation of dissolved organic species, consistent with preferential stabilization of reduced carbon species at the solid–liquid interface, while inorganic carbon concentrations decrease significantly between 24 and 48 h of irradiation, a trend supported by both classical statistics and Bayesian inference (posterior probability *P* ≈ 0.97) and plausibly associated with progressive bicarbonate consumption through surface-mediated pathways. Thermal regeneration experiments further demonstrate partial reversibility of the irradiation-induced spectral features, supporting their surface-bound nature. Taken together, these results establish CeO_2_–SnO_2_ hybrids as useful model systems for fundamental studies of bicarbonate-to-formate-like surface processes and highlight the value of probabilistic and unsupervised analytical frameworks for resolving subtle, irradiation-driven interfacial transformations.

## Graphical abstract

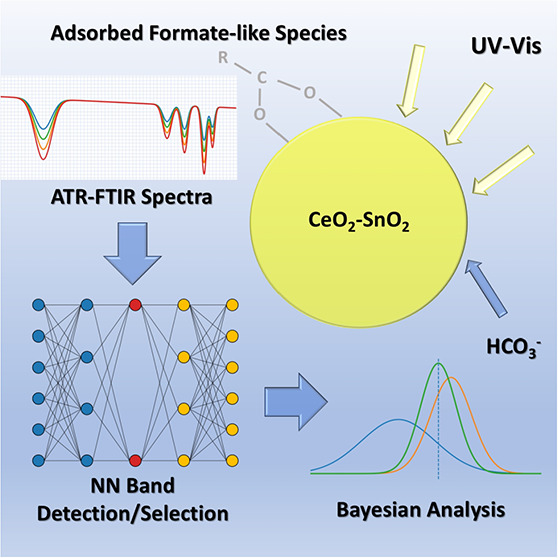

## Introduction

Greenhouse gases, particularly carbon dioxide (CO_2_), are driving a global climate crisis due to their rapidly increasing atmospheric concentrations.^1^ CO_2_ is a major contributor to greenhouse gas emissions, accounting for approximately 33 Gt released in 2021, with levels projected to continue rising through 2050.^2^ Atmospheric CO_2_ concentrations are now at their highest in the past 800,000 years,^3^ recently surpassing 400 ppm,^2,4–6^ and could reach 790 ppm by 2100 if current emission trends persist.^2^ To limit global temperature increases to the 1.5–2 °C maximum stipulated in the 2015 Paris Agreement, CO_2_ emissions must reach net-zero between 2040 and 2060.^1^

Addressing this challenge requires the development of sustainable technologies for carbon capture, utilization, and storage (CCUS).^5^ Among various CCUS strategies, photocatalytic CO_2_ reduction has emerged as a promising approach,^7–9^ mimicking natural photosynthesis to convert CO_2_ into value-added chemicals and store renewable energy in chemical form.^7^ However, prior to conversion, carbon dioxide obviously needs to be captured and delivered to catalytic systems in order to react and get transformed into value-added products. Direct utilization of gaseous CO_2_ in catalysis presents difficulties, such as poor contact between CO_2_ and the catalyst surface, and gas–liquid–solid mass transfer limitations due to the low solubility of CO_2_ in the aqueous phase, thus limiting selectivity.^7,9^ Furthermore, the release of captured CO_2_, usually stored in alkaline aqueous matrices, is energy-intensive, and there is industrial interest in avoiding it.^10^

An ideal alternative to direct CO_2_ utilization is to deliver it to catalytic systems as aqueous forms of CO_2_ ^7^–bicarbonate (HCO_3_^–^) and carbonate (CO_3_^2–^)–which can be easily obtained by capturing carbon dioxide in alkaline solutions^5,7^ as shown in eq 1

1 $${\mathrm{CO}}_{2\left(g\right)}+2{\mathrm{OH}}_{\left(\mathrm{aq}\right)}^{-}⇄{\mathrm{HCO}}_{3(\mathrm{aq})}^{-}+{\mathrm{OH}}_{\left(\mathrm{aq}\right)}^{-}⇄{\mathrm{CO}}_{3(\mathrm{aq)}}^{2-}+H_{2}O_{\left(l\right)}$$

Another abundant and sustainable source of bicarbonate is seawater, where less than 1% of the total dissolved inorganic carbon exists as un-ionized CO_2_; in fact, the world’s oceans function as a vast dilute solution (∼2 mmol/kg) of sodium bicarbonate.^11^ Regardless of the source, using bicarbonate or carbonate solutions in industrial processes eliminates the need for the energy-intensive CO_2_ liberation step, enabling more direct and efficient carbon utilization without additional physicochemical modification.

Various materials and technologies have been explored for the reduction of CO_2_ and its dissolved forms, including bicarbonate. Among these, the electrocatalytic conversion of both gaseous CO_2_ and aqueous CO_2_ (bicarbonate) using Sn-based materials has become a widely investigated strategy for this class of materials.^3,5,6,12–50^ Tin-based materials are particularly well-known for their high selectivity toward the formation of formic acid during both CO_2_ and bicarbonate electroreduction,^3,13,15,16,19,23–25,29,31–33,36,40,44,46,47,49^ making them highly desirable for formic acid production. In contrast, cerium-based materials have established a strong track record as photocatalysts for the reduction of CO_2_ and bicarbonate,^51–66^ although they typically produce methane, methanol, or CO rather than formate or formic acid. Given these complementary attributes, it is reasonable to hypothesize that a CeO_2_–SnO_2_ hybrid could exhibit photocatalytic activity for the conversion of bicarbonate into formate. This hypothesis forms the rationale behind our approach.

Motivated by this rationale, we further characterized a CeO_2_–SnO_2_ hybrid synthesized via a greener, solid-state route^67^ and investigated its ability to drive visible-light assisted conversion of aqueous bicarbonate into formate-like species at a fundamental, proof-of-concept level. To the best of our knowledge, this is the first demonstration of UV–visible-light assisted conversion of bicarbonate (aqueous CO_2_) into organic molecules by a CeO_2_–SnO_2_ hybrid. Previously, Liang et al.^12^ reported the electrocatalytic reduction of gaseous CO_2_ to formic acid using SnO_2_ supported on CeO_2_, but their system differs fundamentally from ours in both material architecture and mechanistic pathway. Unlike their supported material, our material is a true CeO_2_–SnO_2_ hybrid with intimate phase integration.^67^ More importantly, our approach relies on photogenerated electrons for CO_2_ (in its aqueous form of bicarbonate) reduction, rather than direct electron transfer via electrolysis. Finally, while Liang et al. employed gaseous CO_2_ as the substrate, our system utilizes bicarbonate (HCO_3_^–^) in aqueous solution, which is a more practical and environmentally relevant form of dissolved CO_2_.

## Materials and Methods

### Reagents

Tin­(II) chloride dihydrate (SnCl_2_·2H_2_O, 99%) was obtained from Synth (Brazil); cerium­(III) nitrate hexahydrate (Ce­(NO_3_)_3_·6H_2_O, 99%) was purchased from Neon Química (Brazil). Granular activated charcoal and sodium bicarbonate (99%) were acquired from Dinâmica Química (Brazil). Commercial tin dioxide (SnO_2_) was obtained from Faferia Cerâmica (Brazil).

### Synthesis of the Hybrid Material

The material was synthesized as previously described.^67^ Briefly, tin­(II) chloride dihydrate and cerium­(III) nitrate hexahydrate were mixed with granular activated charcoal and ground into a dry paste, microwaved for 30 min, and calcined at 650 °C for 4 h. The resulting crystalline solid was used directly in the catalytic experiments.

### Characterization

The material has been previously and comprehensively characterized by UV–Vis diffuse reflectance spectroscopy, X-ray diffraction (XRD), X-ray photoelectron spectroscopy (XPS), and scanning electron microscopy with energy dispersive spectroscopy (SEM/EDS).^67^ Additional characterization performed in the present work included prereaction transmission electron microscopy (TEM), zeta potential analysis, and pre- and postreaction attenuated total reflection Fourier transform infrared spectroscopy (ATR-FTIR). Prereaction TEM was carried out on a JEOL 1400FLASH (JEOL Ltd., Japan) operating at 120 kV. Micrographs were acquired for particle-size estimation and morphology assessment. TEM particle sizes were extracted from 41 micrographs using an automated image-processing pipeline implemented in Python. Each image was converted to grayscale, corrected for the scale bar, locally contrast-enhanced (CLAHE), and Gaussian-smoothed prior to Otsu thresholding. Morphological filtering was applied to remove small objects and fill internal holes, producing clean binary masks of individual nanoparticles. Particle diameters were obtained from their equivalent-circle diameters using connected-component analysis and converted to nanometers using the calibrated scale (0.36 nm px^–1^). Diameters outside the first–99th percentile were removed to suppress segmentation outliers. All diameter data from the 41 images were pooled into a single data set and fitted to a log-normal distribution using maximum-likelihood estimation, from which the mean, median, mode, and standard deviation of the log-normal particle-size distribution were computed.

Pre- and postreaction characterization was conducted by ATR-FTIR using a PerkinElmer Frontier MIR spectrometer (PerkinElmer Inc., USA) equipped with a universal ATR accessory. ATR-FTIR spectra were collected after the irradiation experiments following separation of the solid by filtration and washing with deionized water (3 × 30 mL), followed by drying at 35 °C overnight. For comparison, the ATR-FTIR spectrum of the air-exposed hybrid and of the physical mixture of the parent oxides were also recorded. For each irradiation condition, ATR-FTIR measurements were performed on the material recovered from a single experimental run. Thus, each spectrum represents the surface state of the material after one independently conducted photocatalytic experiment rather than the mean of multiple spectroscopic replicates.

Prereaction zeta potential analysis was carried out in a Litesizer DLS 700 (Anton Paar GmbH, Austria) in a 0.1% m/v suspension of the hybrid material in deionized water (pH = 7) that had been sonicated for 10 min prior to analysis.

### Photoconversion and Control Experiments

Photoconversion experiments were carried out in a custom-built batch reactor. The exterior of the reactor was temperature-controlled using a water bath maintained at 5 °C. Illumination was supplied by a 400 W medium-pressure mercury vapor lamp (Philips, Brazil) with the outer bulb removed to directly expose the arc tube. To ensure that only UV and visible wavelengths reached the hybrid material, the lamp output was directed through an integrated water filter connected to a circulating ultrathermostatic bath (Marconi, Brazil) maintained at 2 °C.

The experiments were carried out using 0.1 mol·L^–1^ aqueous sodium bicarbonate solutions (pH = 8.0–8.3) containing 0.1% w/v of the suspended CeO_2_–SnO_2_ hybrid. Irradiation times of 6, 24, and 48 h were investigated, with all experiments performed in triplicate. A dark control was conducted for 48 h to assess nonphotochemical background reactions. A further 48 h control irradiation was conducted using the physical mixture of the parent oxides in the same CeO_2_/SnO_2_ ratio (0.568:0.432) examined in our previous work.^67^

Total organic carbon (TOC) and inorganic carbon (IC) measurements were carried out using a Multi N/C 2100 S carbon analyzer (Analytik Jena GmbH + Co. KG, Germany). Analyses were performed on the decanted liquid phase of the reaction mixture, corresponding to the bicarbonate-containing aqueous solution in contact with the suspended hybrid material. Samples were collected from experiments irradiated for 24 and 48 h, comprising three independent samples for each irradiation time.

Measurements of IC and TOC were not performed for the 6 h irradiation condition to minimize contributions from early stage removal or redistribution of adventitious, surface-bound carbon species accumulated during air exposure. Under these conditions, changes in TOC would primarily reflect desorption or oxidation of pre-existing surface contaminants rather than net conversion of dissolved inorganic carbon. Carbon measurements were therefore restricted to the 24 and 48 h irradiation time points, where the influence of such surface-bound impurities is minimized and the measured trends more reliably reflect irradiation-dependent carbon transformation and inorganic carbon consumption.

### Bayesian Inference Analysis of ATR-FTIR Data

Bayesian inference was employed to analyze the ATR-FTIR data sets of recovered, dried samples (one per experimental condition) in order to quantify spectral uncertainty under small-sample conditions typical of surface-sensitive FTIR measurements. Unlike classical statistical approaches, which rely on Gaussian error propagation and multiple replicates, Bayesian inference treats model parameters as probability distributions and allows uncertainty to be propagated directly through the analysis. Previous studies have shown that Bayesian approaches provide reliable uncertainty estimates in small-sample chemical data sets and remain robust when measurement noise deviates from normality.^68–70^

To minimize user-defined bias in spectral interpretation, FTIR spectra were processed using a neural-network–assisted analysis workflow. Raw transmittance spectra were converted to absorbance and interpolated onto a unified 650–3050 cm^–1^ wavenumber grid for analysis. A sparsity-constrained convolutional denoising autoencoder was trained to reconstruct the spectra from a low-dimensional latent representation while Gaussian noise injection and L1 regularization promoted a compact and noise-robust encoding of the spectral manifold. After training, gradient-based saliency maps (∂loss/∂input) were computed to quantify the contribution of each wavenumber to reconstruction accuracy. Normalization and smoothing of this saliency distribution yielded a continuous relevance field *P*(*ν̃*), providing a data-driven estimate of spectral information content across the full wavenumber range.

For quantitative analysis, literature-assigned vibrational bands associated with surface formate and bicarbonate species were first defined using previously reported infrared assignments (Tables 1 and 2).^71–73^ Regions known to overlap with intense IR-active water modes^7,74,75^ were excluded prior to analysis. Within each remaining literature-defined band interval, neural-network relevance was compared with the surrounding spectral background, and an ensemble of unsupervised thresholding criteria (Otsu thresholding, *k*-means clustering, Gaussian mixture modeling, and curvature-based knee detection) was used to retain only vibrational bands exhibiting statistically significant spectral relevance. Importantly, the neural-network output does not define new vibrational bands or modify spectral boundaries; rather, it functions solely as a data-driven filter that selects chemically meaningful, literature-assigned bands while suppressing noise-dominated spectral regions.

**Table 1:** Literature-Assigned Infrared Vibrational Bands Associated with Formate Species Adsorbed on Ceria Surfaces, Including Carboxylate Stretching Modes and C–H Bending and Stretching Vibrations

IR adsorbed formate band^71,72^	wavenumber range (cm^–1^)
Bending of O–C–O group, ν_b_(OCO)	630–710
Out-of-plane C–H bending, π(H–C)	930–1000
C–H deformation/bending, δ(CH)	740–1350
Asymmetric stretch of carboxylate group, ν_as_(COO^–^)	1190–1540
Symmetric stretch of carboxylate group, ν_s_(COO^–^)	960–1330
C–H stretch, ν(CH)	2360–2900

**Table 2:** Literature-Assigned Infrared Vibrational Bands Associated with Bicarbonate Species Adsorbed on Ceria Surfaces, Including Stretching and Bending Modes of O–H, C–O, and O–C–O Groups

IR adsorbed bicarbonate band^73^	wavenumber range (cm^–1^)
Stretching of O–H group, ν(OH)	3663–3710
Asymmetric stretching of C=O group, ν(CO)	1597–1632
Symmetric stretching of C–O group, ν_s_(CO)	1332–1408
Bending of C–O–H group, δ(COH)	1173–1192
Secondary stretch of C–O group, ν(C–O)	999–1017
In-plane bending of O–C–O group, δ(OCO)	772–788

Band intensities were subsequently obtained by numerical integration of the baseline-corrected absorbance within the selected wavenumber intervals. To avoid double counting in partially overlapping spectral regions, mutually exclusive union masks were constructed for each chemical family so that absorbance contributions assigned to formate and bicarbonate bands were integrated independently. This overlap-removal procedure ensures mutually exclusive band areas while remaining consistent with established vibrational assignments.

The resulting NN-selected band areas were analyzed using a nonhierarchical Bayesian model implemented in *PyMC* with the *NumPyro* NUTS (No–U-Turn Sampler).^76^ Because integrated FTIR band intensities are strictly positive and typically right-skewed, all areas were log-transformed and standardized prior to modeling. On this transformed scale, each observation was modeled using a Student-t likelihood *y*_*i*_ ∼ Student-*t*_ν=7_(μ_*i*_,σ), with weakly informative priors μ_*i*_ ∼ *N*(0,1) for each sample-specific mean and σ ∼ HalfNormal(1) for the shared residual scale.

Posterior inference was performed using Hamiltonian Monte Carlo with four NUTS chains, each comprising 3,000 warm-up iterations followed by 6,000 sampling iterations, yielding 36,000 posterior draws in total. The target acceptance probability was set to 0.99. Posterior samples were used to compute pairwise probability-of-superiority matrices *P*(μ_*i*_ > μ_*j*_), providing direct probabilistic comparisons of formate- and bicarbonate-associated band intensities across experimental conditions. In contrast to classical hypothesis tests, which yield only binary accept–reject outcomes, the Bayesian framework provides continuous probability estimates while propagating uncertainty arising from spectral noise, band selection, numerical integration, and the statistical model itself.

A hierarchical model was not employed because the data set consists of independent condition-level observations rather than nested replicate measurements. Each integrated band area therefore represents an independent catalyst preparation and FTIR measurement rather than repeated spectra from the same sample, and introducing hierarchical variance components would not be identifiable with the available sample size. A Student-t likelihood was adopted to provide robustness against potential heavy-tailed noise and occasional outliers commonly observed in surface spectroscopic measurements. The degrees-of-freedom parameter was fixed at ν = 7, which provides moderate tail robustness while remaining close to the Gaussian regime and avoids overparameterization of the model given the limited data set. Robustness checks, including posterior predictive validation and sensitivity analyses to the choice of ν, prior scale, and variance structure, are reported in the Supporting Information (Figures S3 and S4) and confirm that the qualitative ordering of experimental conditions remains stable across reasonable model specifications.

All FTIR preprocessing, neural-network training, saliency analysis, band selection, and Bayesian inference were implemented in Python 3.10 using *NumPy*, *SciPy*, *TensorFlow/Keras*, *PyMC*, *NumPyro*, and *ArviZ* with fixed random seeds to ensure full reproducibility.

### Self-Organizing Map Validation of Bayesian Inference Analysis of FTIR Data

Self-organizing map (SOM) analysis was performed as an independent, unsupervised multivariate validation of the Bayesian inference applied to the FTIR data, with the objective of assessing whether the spectra differed with respect to formate-related vibrational features. FTIR spectra were converted to absorbance, interpolated onto a common wavenumber grid spanning 650–4000 cm^–1^ (corresponding to the full ATR-FTIR spectral range), and preprocessed using standard normal variate (SNV) normalization followed by a Savitzky–Golay first-derivative filter, as implemented in *NumPy* and *SciPy*. The SOM was trained on the full preprocessed spectral data set using the *MiniSom* Python package, projecting the high-dimensional FTIR data onto a two-dimensional lattice while preserving topological relationships among spectra without the use of predefined classes or response variables. The optimal number of SOM prototype clusters was determined automatically using an internal cluster validity criterion, avoiding user-defined selection of cluster number. Unsupervised clustering of SOM neuron weight vectors was then carried out using *scikit-learn*. To interrogate the chemical origin of spectral differentiation, a formate-specific component plane was constructed by averaging SOM neuron weight vectors over literature-assigned formate vibrational bands, excluding regions overlapping with water absorption bands as previously defined. Sample positions (best-matching units) and clustering behavior were evaluated relative to this component plane to determine whether sample separation coincided with variations in formate-related spectral features. All data handling, analysis, and visualization were carried out using the Python scientific computing stack, ensuring full reproducibility of the workflow.

### Classical and Bayesian Inference Analysis of Inorganic Carbon Results

Inorganic carbon (IC) data were analyzed using both classical hypothesis-testing and Bayesian probabilistic frameworks to ensure robust inference under small-sample conditions. Prior to classical analysis, the distributional assumptions required for parametric testing were evaluated; when normality criteria would be satisfied, differences between irradiation times would be assessed using one-way analysis of variance (ANOVA), whereas in cases where these assumptions would not be met, a nonparametric Kruskal–Wallis test would be applied. This decision process was implemented in an adaptive analysis pipeline, allowing objective selection of the appropriate statistical test based on the data rather than a priori preference. In parallel, Bayesian modeling of the IC measurements was performed using a Student-t likelihood to accommodate potential deviations from normality, with posterior inference obtained from four Hamiltonian Monte Carlo NUTS chains using 3,000 warm-up iterations followed by 6,000 draws per chain and a target acceptance probability of 0.99. This approach yielded full posterior distributions for group means and associated uncertainties. Bayesian inference provided complementary information to the classical tests by quantifying the probability of differences between irradiation times and enabling direct probabilistic comparisons, thereby reinforcing the conclusions drawn from the classical analysis while avoiding reliance on asymptotic assumptions.

All computations were performed in Python using *NumPy*, *SciPy*, *PyMC*, *ArviZ*, and *NumPyro* with fixed random seeds for full reproducibility.

### Surface Regeneration Tests

Thermal regeneration of surface-bound carbon species was carried out on the CeO_2_–SnO_2_ hybrid following 48 h of irradiation in bicarbonate-containing aqueous solution. After irradiation, the solid was recovered by filtration, thoroughly washed with deionized water (3 × 30 mL), and dried at 35 °C overnight. The dried material was then thermally treated in air at 200 °C for either 2 or 4 h. After thermal treatment, the samples were allowed to cool to room temperature and were analyzed by ATR-FTIR spectroscopy without any further exposure to bicarbonate solution or light. Formate- and bicarbonate-associated vibrational bands were subsequently quantified using the same overlap-corrected integration and neural-network-assisted band selection procedures described above. Bayesian statistical analysis of the resulting peak areas was conducted using the same inference pipeline and priors as previously described. The target acceptance probability for the No–U-Turn Sampler was increased to 0.999 to improve numerical stability and sampling efficiency for this smaller, lower-variance data set, ensuring robust chain convergence and reliable posterior estimation.

## Results and Discussion

### Transmission Electron Microscopy (TEM) Results

Figure 1 shows representative micrographs of the hybrid material. Figure 1a highlights the apparent structural diversity of the particles, revealing the coexistence of cubic and rod-like morphologies. In ceria-based systems, the presence of nanorods is particularly important, as their more reactive exposed planes are known to enhance catalytic activity relative to bulk CeO_2_.^77^ Considering that the hybrid is most plausibly a tin-doped ceria host structure,^67^ these rod-like crystallites likely correspond to CeO_2_ nanorods with interstitially substituted Sn atoms. If such nanorods are indeed Sn-doped, they would be expected to display an increased ability to convert bicarbonate into reduced organic species. This interpretation is consistent with the irradiation experiments, where only the irradiated hybrid, and not the physical mixture of the parent oxides, promoted formation of formate-like species beyond the control baseline, indicating that the active sites are intrinsic to the possibly doped nanorod (and cubic) architecture rather than arising from simple oxide blending.

**Figure 1:**
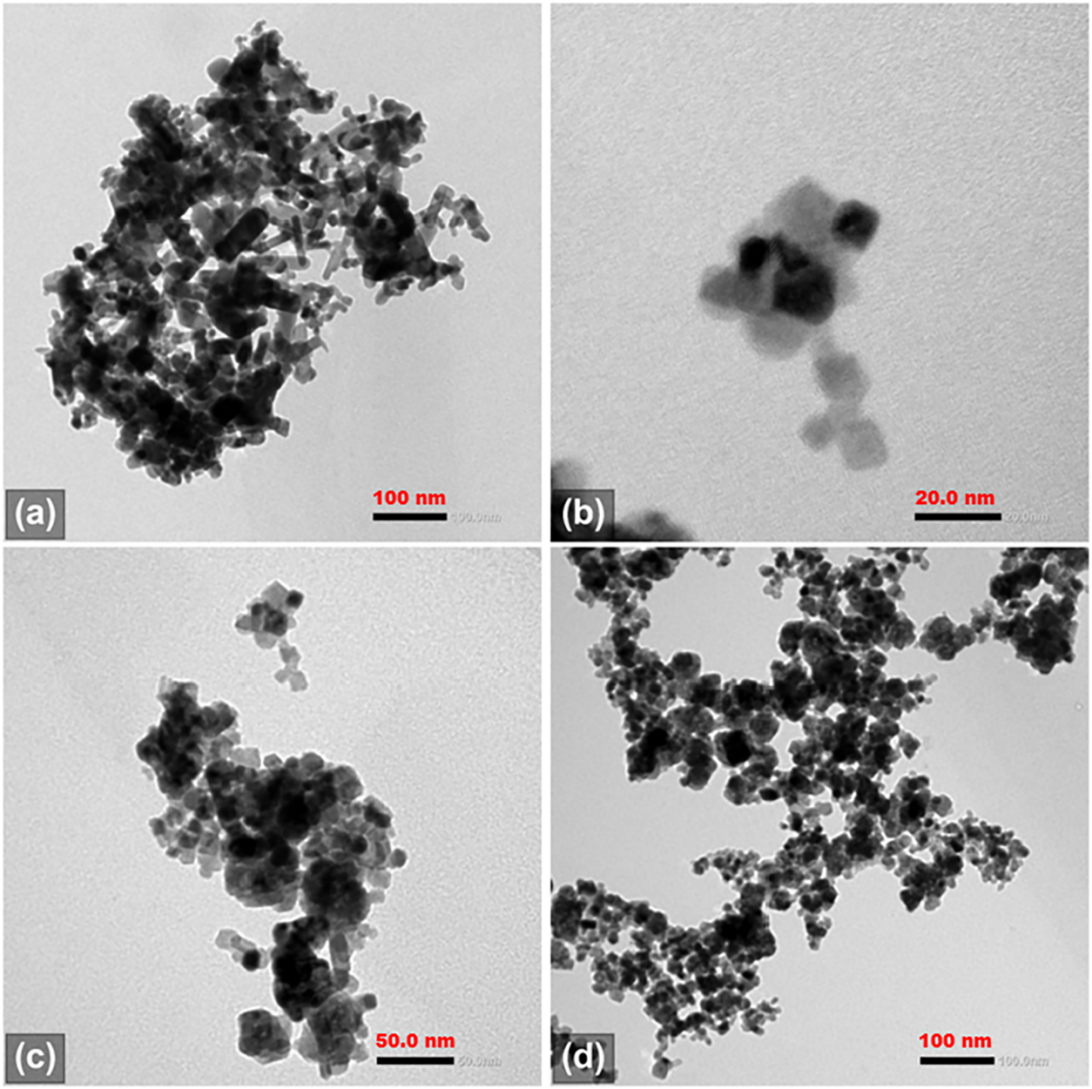
**T**ransmission electron microscopy (TEM) images of the CeO_2_–SnO_2_ hybrid material at different magnifications: (a) low-magnification image showing extended agglomerates formed by nanoscale primary particles (scale bar: 100 nm); (b) high-magnification view highlighting representative particle clusters composed of irregularly shaped nanocrystallites (scale bar: 20 nm); (c) intermediate-magnification image illustrating aggregated particles with characteristic dimensions of several tens of nanometers (scale bar: 50 nm); and (d) low-magnification overview revealing the porous, interconnected nature of the aggregated hybrid oxide (scale bar: 100 nm).

Figure 1b presents a small agglomerate of the hybrid material’s particles, providing a clearer view of their dimensions. Visual inspection indicates that the individual crystallites fall within the ∼5–15 nm size range.

Figure 1c further illustrates the structural diversity of the hybrid material, revealing the presence of larger crystallites (∼40–50 nm in diameter) coexisting with agglomerates composed of much smaller nanoparticles. Figure 1d highlights a fractal-like arrangement of these agglomerates and larger crystals, underscoring the multiscale textural complexity of the material.

Figure 2 shows a representative TEM micrograph of the CeO_2_–SnO_2_ hybrid material, illustrating the aggregation of nanoscale oxide particles on the carbon support.

**Figure 2:**
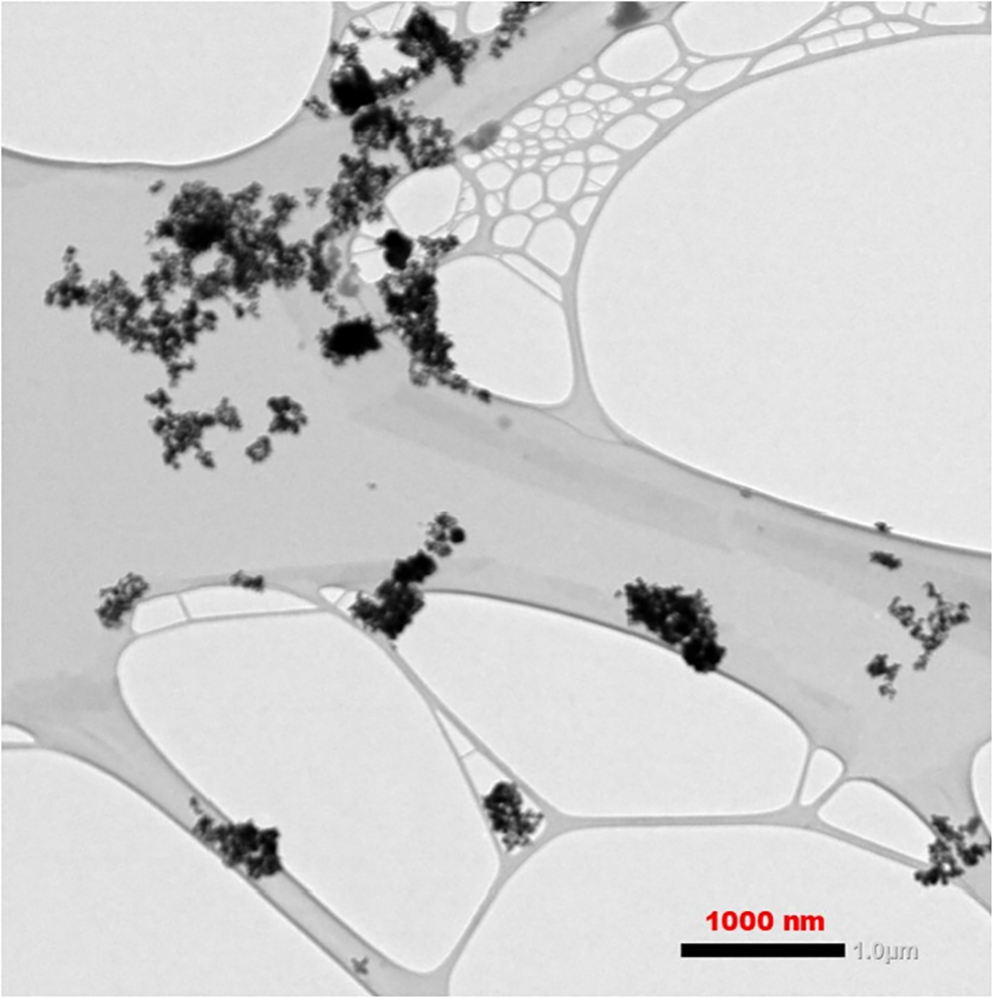
Representative transmission electron microscopy (TEM) micrograph of the CeO_2_–SnO_2_ hybrid material, showing aggregates of nanoscale oxide particles dispersed on the carbon support structure. The scale bar corresponds to 1000 nm.

The TEM micrograph reveals aggregates of nanoscale oxide particles dispersed on the carbon support structure. The darker contrast regions correspond to oxide domains, reflecting their higher electron density relative to the supporting matrix. The clustered morphology suggests that the hybrid material is composed of aggregated nanoscale oxide domains.

Figure 3 presents the particle size distribution for the hybrid in both general and magnified forms. A log-normal fit to the size data (Figure 2a) yielded a mean, median, mode, and standard deviation of 4.04, 3.63, 2.93, and 1.98 nm, respectively. Although some larger particles are evident in the TEM micrographs, the overwhelming majority of crystallites fall within the 2–6 nm range, as shown in Figure 2b–d.

**Figure 3:**
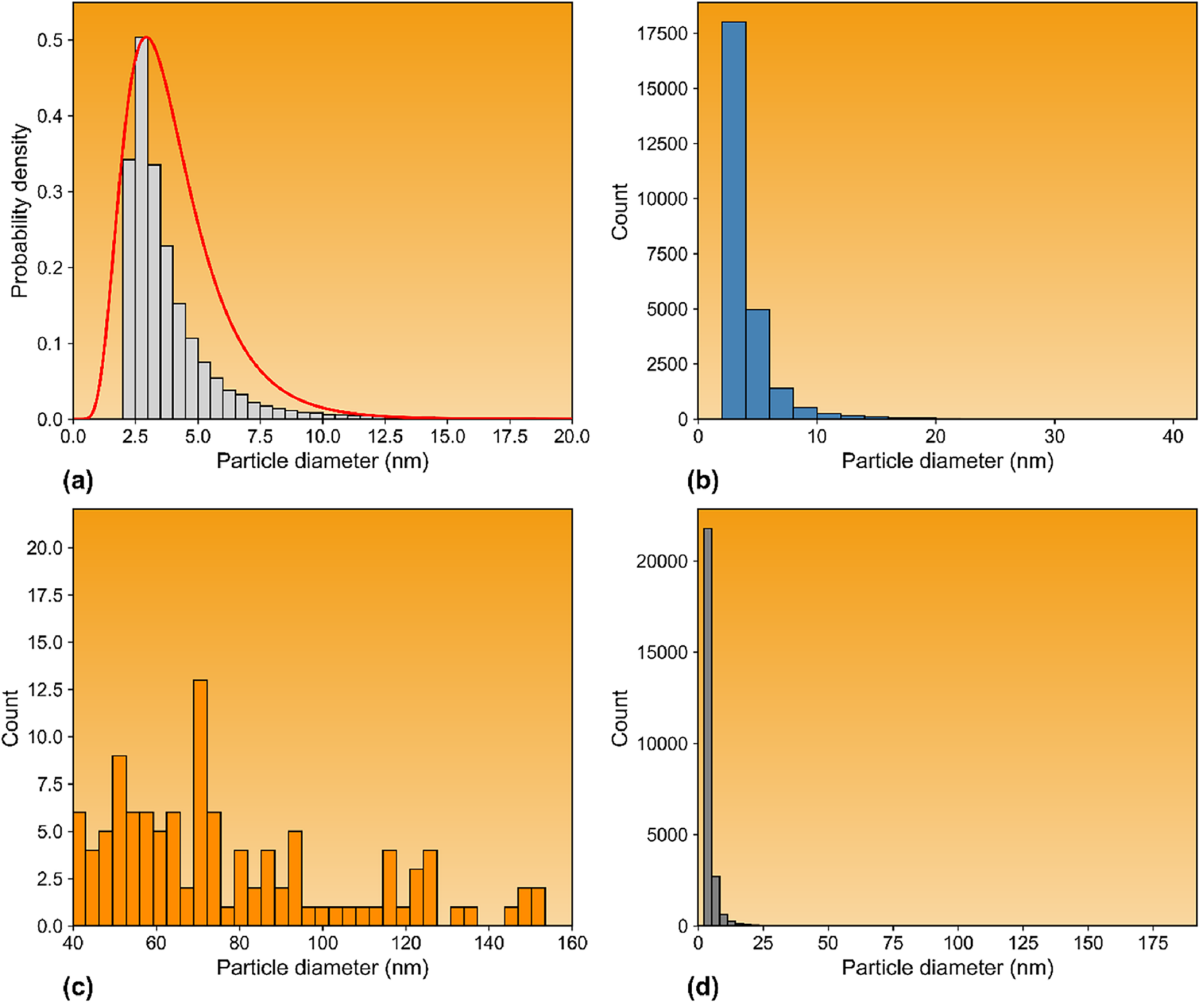
Particle size distributions derived from TEM image analysis of the CeO_2_–SnO_2_ hybrid material at different length scales: (a) probability density distribution computed over the entire measured particle size range, with the solid line representing a log-normal fit; (b) histogram showing particle diameter counts in the nanoscale range (0–40 nm); (c) size distribution of larger agglomerated particles, illustrating the broad dispersion of aggregate dimensions in the tens to hundreds of nanometers range; and (d) combined particle diameter histogram including all measured particles.

Particles in this size regime offer several advantages for photoconversion and photocatalytic applications. Their small dimensions reduce electron–hole recombination rates due to enhanced quantum confinement and shortened carrier migration distances, thereby improving charge separation efficiency.^78,79^ Furthermore, the prevalence of such small crystallites explains the tendency of formate-like species to adsorb onto their surfaces: nanoparticles in the 2–6 nm regime possess high surface-to-volume ratios and correspondingly elevated surface Gibbs free energies, which thermodynamically favor the stabilization of adsorbed species such as formate.

It should be noted that the purpose of the TEM analysis in the present work is to characterize particle morphology and size distribution rather than to establish the structural nature of the CeO_2_–SnO_2_ hybrid. The hybrid structure of this material has been previously demonstrated using complementary techniques including X-ray diffraction, X-ray photoelectron spectroscopy, and UV–Vis diffuse reflectance spectroscopy in our earlier work.^67^ The TEM observations presented here therefore serve primarily to illustrate the nanoscale morphology and aggregation behavior of the particles rather than to distinguish between supported and integrated oxide architectures.

### Zeta Potential Results

Figure 4 shows the zeta potential distribution of the hybrid material’s particles in neutral, aqueous solution:

**Figure 4:**
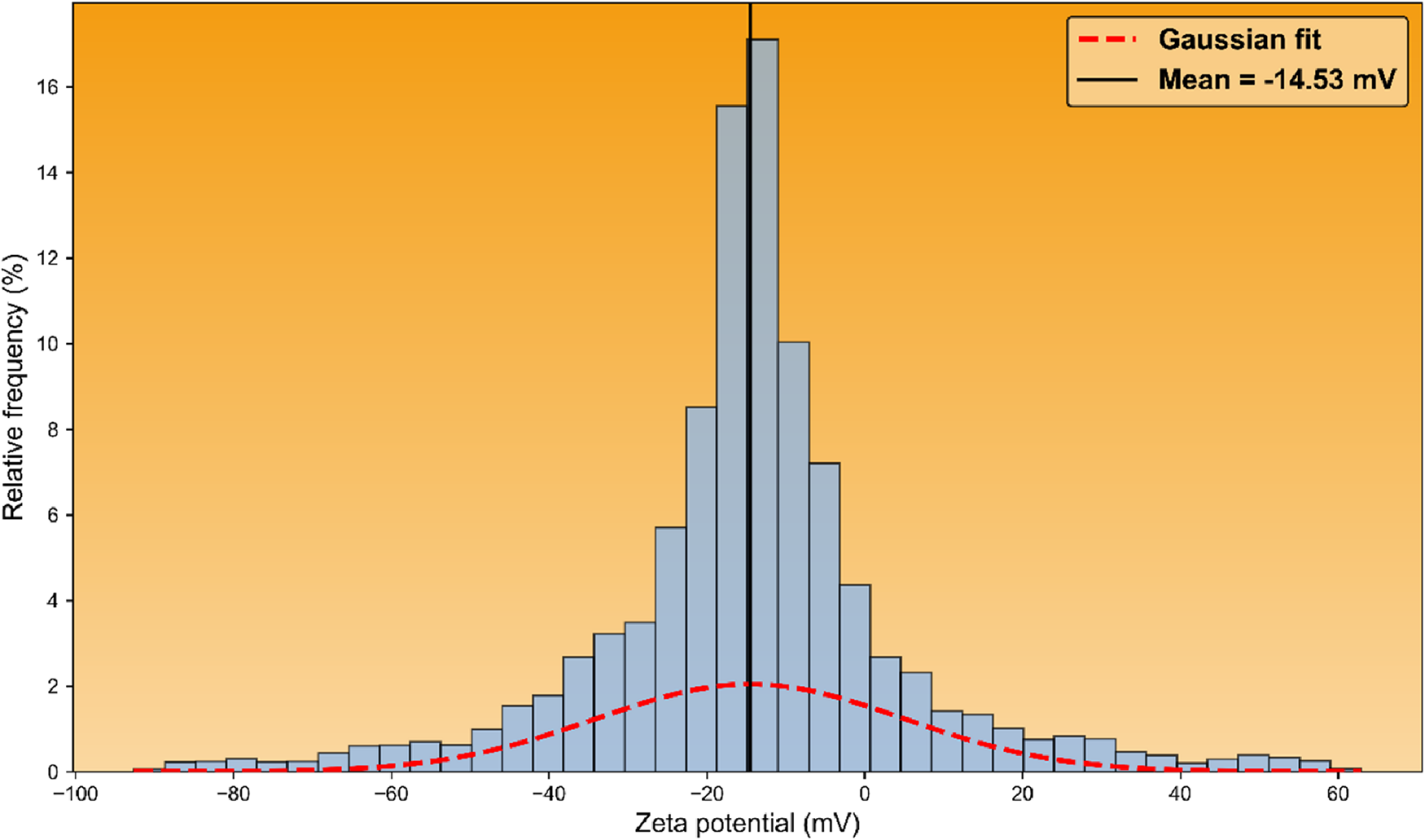
Zeta potential distribution of the CeO_2_–SnO_2_ hybrid material measured in aqueous suspension. The histogram shows the relative frequency (%) of measured zeta potential values, while the dashed curve represents a Gaussian fit to the distribution. The vertical solid line indicates the mean zeta potential value (−14.53 mV).

As shown in Figure 4, the hybrid material’s particles exhibit a Gaussian distribution of zeta potentials centered at −14.53 mV with a relatively broad standard deviation of 19.52 mV, indicating a heterogeneous surface charge population. The particles therefore display both positively and negatively charged domains, which is consistent with surface heterogeneity arising from mixed oxide composition, variable surface hydroxylation, and dynamic adsorption of electrolyte species. The predominance of negative values suggests that, under the measurement conditions, deprotonated surface sites and anion adsorption contribute significantly to the overall electrokinetic behavior, while the presence of a positive tail likely reflects the coexistence of locally protonated surface regions.

Based on the mean and standard deviation of the Gaussian zeta-potential distribution, approximately 66% of the particle population is expected to exhibit absolute zeta potential values in the range of 10–50 mV, corresponding to moderate to high electrostatic stabilization and suggesting that a substantial fraction of the material remains stably dispersed under the measurement conditions.^80^

Under mildly alkaline conditions such as pH 8–8.3, the zeta potential distribution of the hybrid particles would be expected to shift toward more negative values relative to near-neutral pH, reflecting progressive deprotonation of surface hydroxyl groups and enhanced association of bicarbonate and carbonate species at the particle–solution interface. This shift would likely reduce the fraction of positively charged particles and compress the positive tail of the distribution, while increasing the prevalence of negatively charged surface domains. As a result, the electrokinetic behavior of the dispersion would become more uniformly negative, consistent with alkaline, bicarbonate-buffered conditions.

### FTIR Results

Figure 5 shows the normalized FTIR spectra of the hybrid material and the physical mixture of the parent oxides pre- and post- irradiation and dark control experiments (dried, recovered samples; one sample per experimental condition). Figure 5a shows the evolution of the FTIR spectra of the hybrid material (HM) prior to irradiation and after 6, 24, and 48 h of exposure to the bicarbonate-containing reaction medium. After 6 h of irradiation, the spectrum is dominated by broad absorptions centered at approximately 3200 and 1630 cm^–1^, which are assigned to O–H stretching, ν­(OH), and H–O–H bending, δ­(HOH), modes of hydrogen-bonded, molecularly adsorbed water. Water is expected to adsorb to deprotonated surface hydroxyl groups of metal oxides through hydrogen bonding interactions,^81^ which is consistent with the predominantly negative zeta potential measured for the particles. Contributions from adsorbed bicarbonate species may also be present in this region, given the substantial spectral overlap between water-related vibrations and bicarbonate-associated bands under hydrated conditions.

**Figure 5:**
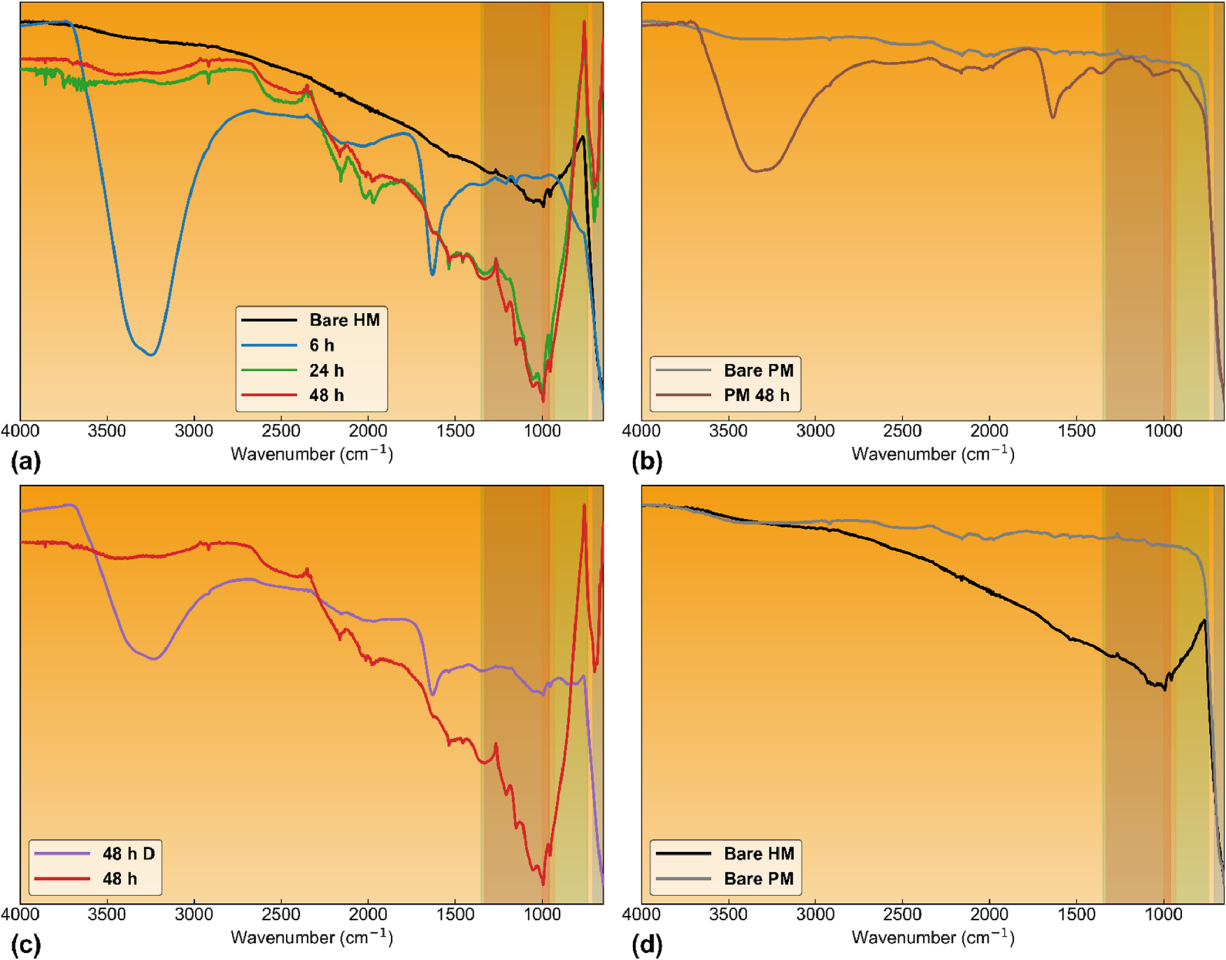
ATR-FTIR spectra of the CeO_2_–SnO_2_ hybrid material and the physical mixture (PM) under different treatment conditions: (a) FTIR spectra of the hybrid material before irradiation (bare HM) and after 6, 24, and 48 h of visible-light irradiation in bicarbonate-containing aqueous solution; (b) FTIR spectra of the physical mixture before irradiation (bare PM) and after 48 h of irradiation under identical conditions; (c) comparison of the irradiated hybrid material after 48 h with the corresponding dark control (48 h D); and (d) FTIR spectra of the bare hybrid material and bare physical mixture prior to irradiation. Spectra are shown after baseline correction and normalization for clarity. All spectra correspond to recovered, washed and dried material from aqueous bicarbonate solutions. Colored shaded regions indicate the wavenumber ranges used for numerical integration of formate-related vibrational bands.

With increasing irradiation time to 24 and 48 h, bands characteristic of surface-bound formate progressively emerge. These include overlapping contributions in the 740–1350 cm^–1^ region from δ­(CH) deformations and O–C–O bending modes, as well as ν_s_(COO^–^) and ν_as_(COO^–^) stretches in the 960–1540 cm^–1^ range. A distinct, albeit weak, band centered near ∼2900 cm^–1^ corresponds to the C–H stretching mode, ν­(CH), which is also characteristic of formate species.^7^ The concurrent growth of these features suggests the progressive buildup of surface-bound formate-like species under irradiation. No pronounced spectral features attributable to molecular water or bicarbonate are observed in the analyzed regions for the hybrid material samples irradiated for 24 and 48 h, indicating that their contribution to the observed bands is minor. A weak spectral feature is observed in the 2300–2350 cm^–1^ region, which may correspond to residual atmospheric CO_2_ or weakly adsorbed CO_2_ species.^82^

A pronounced band near ∼1600 cm^–1^ is observed in the 6 h spectrum and is assigned to bicarbonate-related vibrational modes on the catalyst surface. At this irradiation time, the feature appears relatively sharp and well-defined, which makes it visually dominant within this spectral region. At longer irradiation times (24 and 48 h), the spectral envelope around 1600 cm^–1^ becomes broader and more structured, likely reflecting overlapping contributions from multiple carbonate- and formate-related vibrations. As a result, the distinct peak observed at 6 h becomes less visually pronounced even though bicarbonate-related contributions remain present. The apparent differences in signal-to-noise between spectra primarily arise from variations in band definition rather than measurement conditions, as all spectra were collected under identical instrumental parameters.

Figure 5b compares the FTIR spectra of the physical mixture (PM) of the parent oxides prior to irradiation and after 48 h of irradiation. Following irradiation, the spectrum closely resembles that of the hybrid material irradiated for 6 h (Figure 5a), being dominated by broad O–H stretching and H–O–H bending features characteristic of hydrogen-bonded, molecularly adsorbed water, albeit with lower overall intensity. Contributions from adsorbed bicarbonate species are also likely in this region, owing to substantial overlap between bicarbonate-associated vibrations and water bands under hydrated conditions. These observations indicate that prolonged irradiation primarily induces partial hydration of the oxide surfaces in the physical mixture, with water and bicarbonate plausibly competing for adsorption sites. However, distinct vibrational features attributable to reduced carbon species are not clearly resolved. In particular, carboxylate and C–H bands associated with surface-bound formate remain absent or poorly defined. The predominance of overlapping water- and bicarbonate-related absorptions, together with the lack of identifiable formate signatures, indicates that the photoreactivity of the hybrid toward bicarbonate-to-formate-like species conversion is fundamentally distinct from that of the parent oxides and cannot be explained by simple physical mixing or inherited surface properties.

Figure 5c compares the FTIR spectra of the hybrid material maintained in the dark for 48 h with that of the hybrid irradiated for 48 h under otherwise identical conditions. The dark control exhibits primarily hydration-related features, whereas the irradiated hybrid shows a pronounced enhancement of bands in the 740–1350 and 960–1540 cm^–1^ regions, corresponding to δ­(CH), O–C–O bending, and the ν_s_(COO^–^)/ν_as_(COO^–^) modes characteristic of surface-bound formate. The simultaneous appearance of a weak C–H stretching contribution near ∼2900 cm^–1^ further supports formate-like species accumulation under irradiation.^7^ The absence of comparable carboxylate intensification in the dark sample indicates that irradiation with light, rather than mere contact with bicarbonate solution, is required to promote and stabilize formate species on the hybrid surface.

Interestingly, the ATR-FTIR spectrum obtained after 6 h of irradiation closely resembles that observed after 48 h under dark conditions. This similarity suggests that irradiation accelerates the surface transformations occurring on the CeO_2_–SnO_2_ hybrid. In other words, the surface state reached after prolonged dark exposure appears to be achieved more rapidly under irradiation, consistent with a light-assisted evolution of carbonate- and formate-related surface species at the oxide interface.

Figure 5d compares the FTIR spectra of the bare hybrid material (HM) and the bare physical mixture (PM) prior to irradiation. In both cases, the spectra are dominated by broad, featureless absorptions and exhibit no distinct bands in the carboxylate region (960–1540 cm^–1^) or in the C–H stretching region (∼2900 cm^–1^). The absence of formate- or bicarbonate-related vibrational features indicates that neither parent oxide material appreciably stabilizes carbonate-derived reduced surface intermediates upon exposure to air. This comparison underscores the chemically distinct surface functionality of the hybrid oxide relative to the physical mixture of its parent oxides and provides context for the different spectroscopic responses observed under UV–Vis irradiation in the presence of bicarbonate (Figure 4a–c).

### Results of Bayesian Inference Analysis of FTIR Data

Table 3 shows the integrated total formate-related FTIR band areas for each of the experiments after NN selection and integration. Each area refers to a single FTIR measurement of a single sample.

**Table 3:** Integrated Total Formate Band Areas Obtained from ATR-FTIR Spectra of Control and Irradiated Samples under the Indicated Conditions^a^

sample	conditions	integrated total formate band area, a.u.
Bare PM	Air-exposed physical mixture of parent oxides	0.35
Bare HM	Air-exposed hybrid material	0.63
48 h D	48 h dark experiment, hybrid material suspended in 0.1 M NaHCO_3_	0.77
6 h	6 h irradiation, hybrid material suspended in 0.1 M NaHCO_3_	1.34
24 h	24 h irradiation, hybrid material suspended in 0.1 M NaHCO_3_	8.12
48 h	48 h irradiation, hybrid material suspended in 0.1 M NaHCO_3_	16.77
PM 48 h	48 h irradiation, physical mixture of parent oxides suspended in 0.1 M NaHCO_3_	0.057

a Band areas were calculated by numerical integration over literature-assigned formate vibrational regions (ν_b_(OCO) 630–710 cm^–1^, δ­(CH) 740–1350 cm^–1^, π­(H–C) 930–1000 cm^–1^, and ν_s_(COO) 960–1330 cm^–1^) following baseline correction and exclusion of overlapping bicarbonate contributions, and are reported in arbitrary units (a.u.). The values provide a comparative measure of relative formate-associated surface species across samples and irradiation times rather than absolute surface coverages.

According to Table 3, the irradiated hybrid material exhibits a clear and monotonic increase in formate-associated surface species with irradiation time. After 6 h of irradiation, a measurable but modest formate signal is detected, which increases at 24 h and approximately doubles after 48 h, consistent with progressive accumulation of surface-bound formate-like species under continued light exposure. This time-dependent behavior suggests an association between formate-like species formation and photoassisted surface processes.

In contrast, the hybrid material subjected to 48 h under dark conditions exhibits a substantially lower integrated formate-related signal, comparable in magnitude to that observed for the air-exposed hybrid. The presence of a weak formate-related signal in both dark-treated and air-exposed hybrid samples is attributed to adventitious carboxylate species commonly formed on ceria-based materials upon exposure to ambient CO_2_ and moisture. Ceria surfaces are known to readily adsorb trace carbonate and formate species from air,^83^ which likely leads to the detection of formate-related signals even in the absence of irradiation. The magnitude of this background signal remains nearly constant across dark-treated and air-exposed hybrid samples, indicating that it does not arise from solution-phase bicarbonate conversion but rather from pre-existing surface chemistry.

The higher apparent formate-related FTIR intensity observed for the air-exposed physical mixture compared to the irradiated physical mixture might arise from the different stability of surface carbon species under ambient and aqueous irradiation conditions. Under air exposure, ceria-containing particles readily accumulate weakly bound adventitious carbonate and formate species originating from atmospheric CO_2_ and moisture^83^ as previously mentioned, leading to a measurable signal. Upon suspension in bicarbonate solution and prolonged irradiation, these weakly adsorbed species are likely partially removed or redistributed through equilibration with the aqueous phase and photoassisted surface cleaning. As a result, irradiation does not lead to net formate-like species accumulation on the physical mixture, and the residual surface signal after 48 h remains lower than that of the air-exposed material. This behavior might further indicate that the pronounced formate accumulation observed for the CeO_2_–SnO_2_ hybrid reflects intrinsically distinct chemical and surface properties of the hybrid material, rather than simple inheritance of characteristics from the individual parent oxides.

Bayesian inference analysis based on a framework with Student-t likelihoods rigorously assessed whether the observed differences in integrated band areas exceeded spectral noise and measurement uncertainty. The inference algorithm yielded posterior distributions with effective sample sizes exceeding typical thresholds and *R̂* values equal to unity for all parameters (see Supporting Information, Tables S1–S5), indicating good convergence and robust inference (effective sample size ≥ 400 and *R̂* < 1.01 are commonly considered acceptable diagnostics^84^). Figure 6 shows the probability of superiority (PS) matrix heatmap for the integrated band areas across the experimental systems investigated.

**Figure 6:**
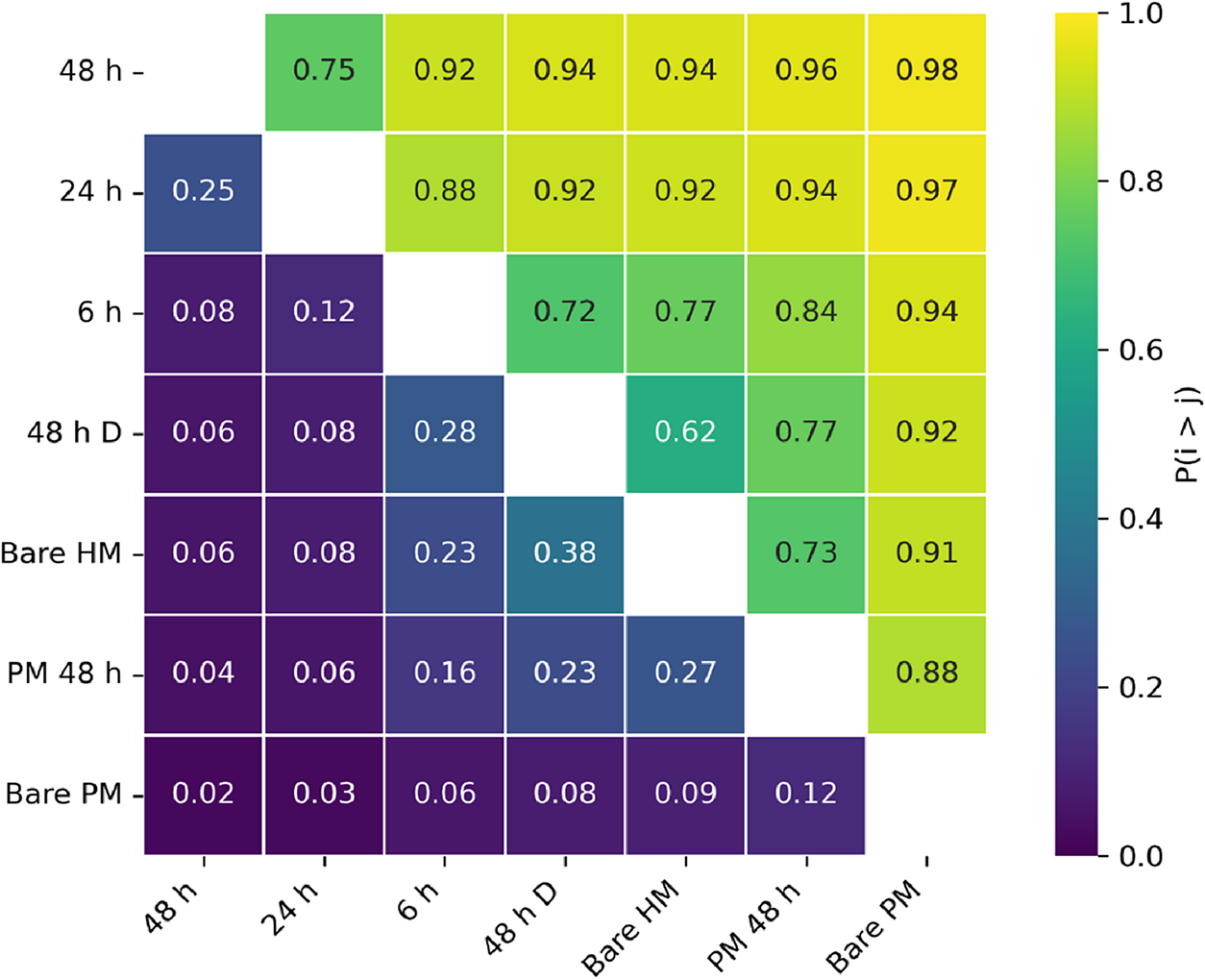
Pairwise probability matrix derived from Bayesian posterior inference of formate-associated FTIR band intensities for all samples. Each cell reports the posterior probability *P*(μ_*i*_ > μ_*j*_) that the mean formate-associated band intens*i*ty of sample *i* exceeds that of sample *j*, based on the full posterior distribution. Higher probabilities (yellow) indicate stronger evidence that one condition exhibits greater formate-associated surface signal than another, whereas values near 0.5 indicate substantial overlap between posterior distributions.

For the formate-associated bands, pairwise posterior probability analysis reveals a clear and internally consistent ordering of surface formate accumulation as a function of irradiation history and material type (Figure 6). The CeO_2_–SnO_2_ hybrid irradiated for 48 h exhibits a very high probability of greater formate-related intensity relative to all other samples, including the 24 and 6 h irradiated hybrids (*P* ≈ 0.75–0.92), as well as all control conditions (*P* ≥ 0.94). Likewise, the 24 and 6 h irradiated hybrid samples display high probabilities of exceeding the dark-treated hybrid, the air-exposed hybrid, and both physical-mixture samples (*P* ≈ 0.72–0.97), confirming a progressive, irradiation-dependent increase in surface formate species. In contrast, the dark-treated and air-exposed hybrid samples occupy an intermediate regime, while the physical mixture under irradiation and the air-exposed physical mixture consistently yield the lowest probabilities across comparisons, indicating minimal formate species accumulation on these surfaces. Collectively, these probabilistic comparisons suggest that sustained formate-like species formation is preferentially associated with irradiation of the hybrid material, rather than with dark conditions or with physical mixtures of the parent oxides under either dark or irradiated conditions.

### SOM Validation of Bayesian Inference Results

As previously mentioned, to independently validate the probabilistic ordering obtained from the Bayesian analysis, a self-organizing map (SOM) was applied to the overlap-corrected FTIR formate-associated band intensities. SOMs provide an unsupervised, topology-preserving projection of multidimensional spectral features and thus allow assessment of whether samples with similar spectral behavior cluster naturally without imposing prior assumptions.

As shown in Figure 7a, the SOM activation map for formate-related features reveals a clear spatial separation between irradiated hybrid samples and control materials. The 48 and 24 h irradiated CeO_2_–SnO_2_ hybrids occupy regions of high activation intensity, indicating strong association with formate-relevant spectral patterns. The 6 h irradiated hybrid, as well as the dark-treated hybrid, air-exposed hybrid, and both physical-mixture samples are located in regions of markedly lower activation, reflecting weak or background-level formate-associated signals.

**Figure 7:**
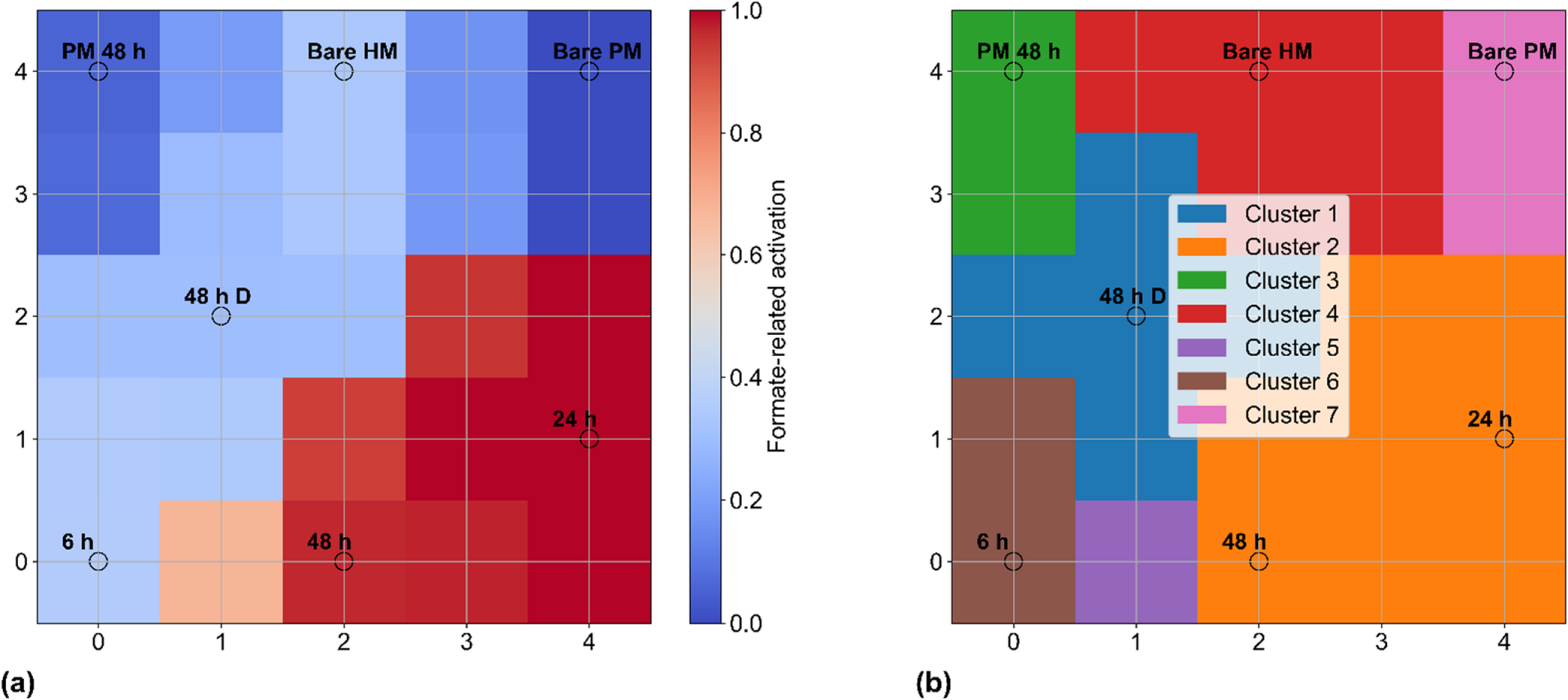
Self-organizing map (SOM) analysis of the FTIR data set highlighting formate-related spectral features and unsupervised sample clustering: (a) formate-specific SOM component plane constructed by averaging SOM neuron weight vectors over literature-assigned formate vibrational bands, with color intensity indicating relative formate-related activation; open circles denote the best-matching units (BMUs) corresponding to each sample; (b) unsupervised clustering of SOM neuron weight vectors based on full preprocessed FTIR spectra, showing cluster assignments independent of band selection. Sample labels indicate the BMU locations for each condition.

The clustering structure extracted from the SOM (Figure 7b) corroborates the Bayesian inference results, with samples segregating into distinct clusters according to irradiation history and material type. Each condition occupies its own cluster except the 24 and 48 h irradiated CeO_2_–SnO_2_ hybrids, which group together in a single region of the map. This joint cluster coincides with the zone of highest formate-related activation in the component plane (Figure 7a), indicating that prolonged irradiation drives the hybrid material toward a common, formate species-enriched surface state. In contrast, the 6 h irradiated hybrid, the dark-treated hybrid (48 h D), the air-exposed hybrid, and both physical-mixture samples (PM 48 h and Bare PM) populate separate clusters located in regions of lower activation, demonstrating that none of the control conditions share the high-formate spectral signature observed for the long-irradiated hybrid samples.

Importantly, although formate-associated bands were included among the SOM input features to visualize which spectral regions activate specific neurons (Figure 7a), this band-weighting step was used only for visualization purposes and did not influence how the SOM formed its clusters. In a self-organizing map, each “neuron” represents a reference spectrum, a prototype vector, that competes during training to best represent a subset of the input spectra, and neighboring neurons adjust together so that the map preserves topological relationships in the data. Because the SOM was trained on the full, unweighted spectral fingerprints, its classification reflects global similarities across all vibrational features rather than any single peak or predefined band group. As a result, the clustering pattern independently reproduces the same qualitative ordering revealed by Bayesian inference of the integrated FTIR band areas, despite relying on entirely different principles. This convergence suggests that the enhancement of formate-related vibrational signatures is a robust, intrinsic property of the irradiated CeO_2_–SnO_2_ hybrid and not an artifact of visualization choices, statistical modeling, background carbonate species, or contributions from the parent oxides.

### Inorganic and Total Organic Carbon (TOC) Results

Table S6 summarizes the inorganic carbon (IC) and total organic carbon (TOC) results for samples of the hybrid material irradiated in the presence of bicarbonate. As shown in Table S6, TOC concentrations for all irradiated samples were below the quantification limit of the analytical method and are therefore reported as zero within experimental uncertainty, indicating the absence of measurable dissolved organic carbon in the bulk aqueous phase during irradiation. This observation is consistent with a surface-mediated transformation pathway in which reduced carbon species are preferentially stabilized at the solid–liquid interface rather than released into solution. Accordingly, the TOC results support an interpretation in which carbon conversion under irradiation is dominated by interfacial reduction and adsorption processes, rather than homogeneous organic carbon formation in the bulk aqueous phase.

The measured inorganic carbon values decrease systematically relative to the common starting point of 1200 mg/L, reaching approximately 1040–1090 mg L^–1^ after 24 h and 900–1020 mg L^–1^ after 48 h of irradiation. Because this decrease is not accompanied by any detectable increase in dissolved organic carbon, the loss of inorganic carbon cannot be attributed to homogeneous conversion into bulk-phase organic species. Instead, these results indicate that inorganic carbon is possibly progressively removed from the aqueous phase through adsorption and surface-mediated reduction on the hybrid material, yielding particle-bound reduced carbon species that remain sequestered at the solid–liquid interface.

Statistical analysis confirmed that inorganic carbon (IC) concentrations exhibited a decrease with irradiation time. Bayesian analysis using a Student-t likelihood yielded posterior mean IC values of 1073 ± 40 mg L^–1^ for the 24 h samples and 964 ± 43 mg L^–1^ for the 48 h samples, with acceptable convergence diagnostics (*R̂* ≈ 1.00 and effective sample sizes exceeding 8,000 for all parameters). The posterior probability that IC at 24 h exceeds that at 48 h was 0.97, providing strong probabilistic evidence for a systematic reduction in inorganic carbon with extended irradiation.

Classical statistical analysis supported this conclusion. Shapiro–Wilk tests indicated non-normality for the 24 h data (*p* < 0.001) but not for the 48 h data (*p* = 0.81); consequently, a nonparametric Kruskal–Wallis test was selected by the adaptive analysis pipeline. The Kruskal–Wallis test revealed a statistically significant difference between irradiation times (*H* = 3.97, *p* = 0.046), confirming that the observed decrease in IC with irradiation time is unlikely to arise from random variability alone. Taken together, the classical and Bayesian analyses consistently demonstrate a significant and directional reduction in inorganic carbon concentration between 24 and 48 h of irradiation.

Since all irradiated suspensions were prepared with the same initial inorganic carbon concentration (1200 mg. L^–1^, supplied as NaHCO_3_), direct comparison of carbon evolution as a function of irradiation time is possible, allowing estimation of an apparent surface-associated conversion fraction of approximately 10% after 24 h and 20% after 48 h of irradiation. This apparent fraction reflects net removal of inorganic carbon from the aqueous phase and does not represent a true stoichiometric yield or exclusive product formation. This fraction may encompass multiple surface-bound reduced organic species; however, spectroscopic evidence indicates that it is likely dominated by formate rather than longer-chain carboxylic acids or alcohols, as no terminal methyl C–H stretching modes or alcohol O–H stretching bands were observed in the FTIR spectra after either 24 or 48 h of irradiation.

The difference in inorganic carbon observed between the 24 and 48 h irradiation experiments cannot be attributed to increased bicarbonate adsorption on the hybrid material. Overlap-corrected integrated areas associated with bicarbonate-related vibrational features are 5.44 × 10^–4^ and 1.29 × 10^–3^ a.u. for the 24 and 48 h irradiation experiments, respectively, whereas the air-exposed hybrid material exhibits a larger bicarbonate-associated contribution of 1.90 × 10^–3^ a.u. Consistent with this trend, Bayesian inference indicates a high posterior probability that the air-exposed hybrid contains a greater abundance of bicarbonate-related surface features than the irradiated samples (*P* ≈ 0.89 relative to the 24 h sample and *P* ≈ 0.94 relative to the 48 h sample; Figure S1). These results indicate that prolonged irradiation does not lead to bicarbonate accumulation at the surface. Instead, the observed decrease in inorganic carbon with increasing irradiation time is consistent with irradiation-induced redistribution of carbon species toward reduced, surface-associated forms rather than enhanced bicarbonate adsorption.

### Surface Regeneration Test Results

Figure 8 shows the ATR-FTIR of the heat-treated samples (200 °C, 2 and 4 h) compared to the air-exposed hybrid material:

**Figure 8:**
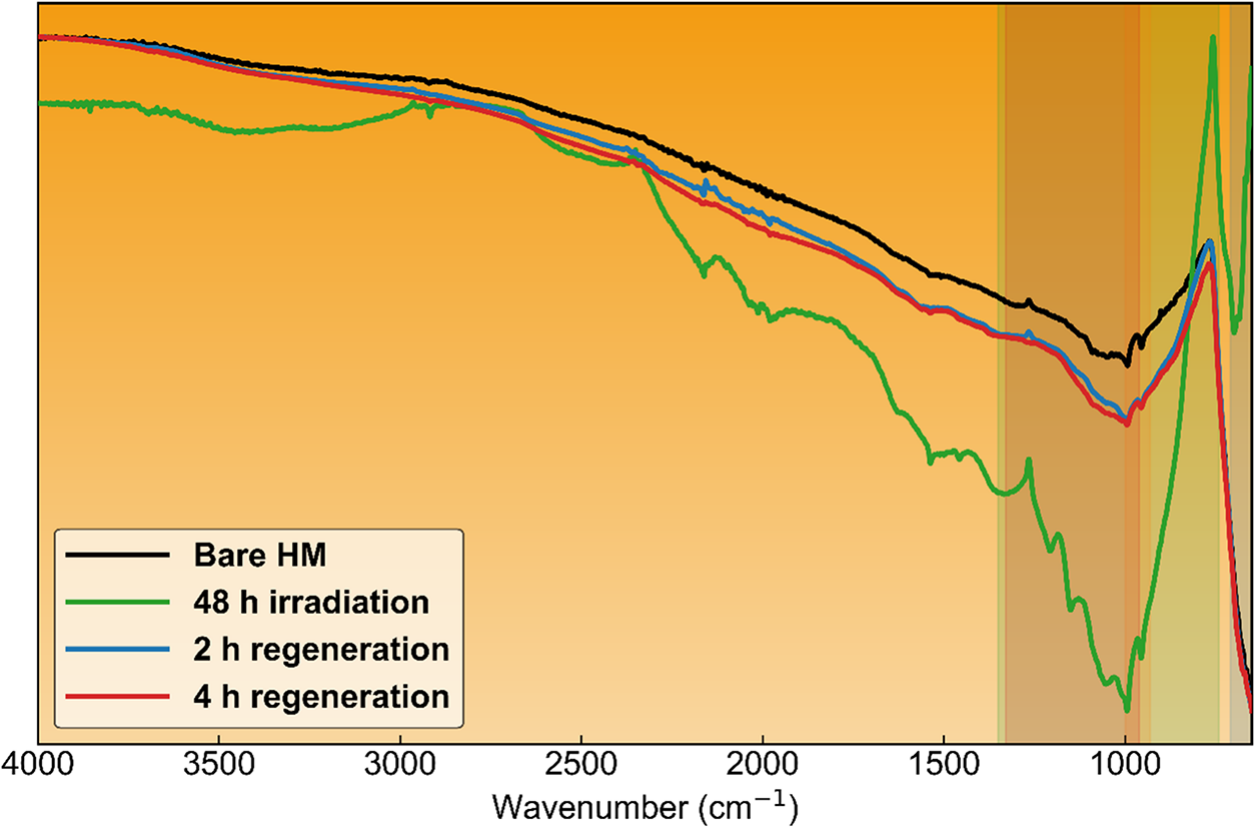
ATR-FTIR spectra of the CeO_2_–SnO_2_ hybrid following irradiation (48 h) and subsequent regeneration treatments (2 and 4 h at 200 °C), shown together with the pristine hybrid material (Bare HM). Spectra are normalized. Colored shaded regions indicate the principal FTIR bands used to guide interpretation of the spectral changes.

Figure 8 provides additional insight into the reversibility and stability of the irradiation-induced surface chemistry of the CeO_2_–SnO_2_ hybrid. After 48 h of irradiation, the ATR-FTIR spectrum differs markedly from that of the air-exposed hybrid across the fingerprint region, consistent with the accumulation of irradiation-induced surface species. Thermal regeneration at 200 °C for 2 and 4 h leads to a partial reversion of the spectra toward the air-exposed hybrid profile, indicating that a substantial fraction of these species is removable under mild treatment. However, regeneration does not result in complete spectral recovery, and residual features persist relative to the air-exposed hybrid, demonstrating that irradiation produces surface-associated species that are only partially reversible under the applied regeneration conditions.

Quantitative analysis of overlap-corrected band areas supports this interpretation. Formate-associated bands remain pronounced after regeneration, with total areas of 1.92 and 1.45 au for the 2 and 4 h treatments, respectively, whereas bicarbonate-associated bands exhibit only modest variation, with total areas of 1.62 × 10^–3^ and 1.29 × 10^–3^ a.u., comparable to that of the air-exposed hybrid (1.91 × 10^–3^ a.u.). These results indicate that regeneration predominantly affects irradiation-induced surface species while leaving the background bicarbonate coverage largely unchanged. The observed partial reversibility is therefore consistent with a condition-dependent surface association rather than irreversible transformation and aligns with the absence of significant accumulation of freely dissolved organic carbon in solution.

Figure S2a,b further contextualize these observations through Bayesian pairwise posterior probability analysis with excellent convergence (*R̂* ≈ 1.00; ESS > 8,000) of the overlap-corrected FTIR band areas. For formate-like features (Figure 8a), both regeneration treatments show a high posterior probability of exceeding the air-exposed hybrid material (Bare HM) (*P* ≈ 0.92–0.93), indicating that formate-related spectral contributions after regeneration cannot be attributed to baseline carbonate adsorption or physical mixing; by contrast, the posterior probabilities distinguishing 2 and 4 h regeneration are low (*P* ≤ 0.39), indicating there is likely no difference between regeneration times regarding residual surface formate-like species. For bicarbonate-like features (Figure 8b), Bayesian posterior probabilities indicate higher bicarbonate-associated band areas for the air-exposed hybrid than for either regenerated sample (*P* ≈ 0.94 and 0.84 relative to 4 and 2 h regeneration, respectively), with the 2 h treatment showing a higher posterior probability of exceeding the 4 h treatment. This ordering is consistent with bicarbonate representing a weakly bound background surface species that is progressively diminished under longer thermal regeneration.

## Conclusions

This work demonstrates that a CeO_2_–SnO_2_ hybrid synthesized via a solid-state route undergoes a distinct, irradiation-dependent evolution of surface chemistry when exposed to bicarbonate-containing aqueous media. ATR-FTIR spectroscopy reveals the progressive emergence of vibrational features consistent with surface-bound formate with increasing irradiation time, whereas dark-treated samples and physical mixtures of the parent oxides remain dominated by overlapping water- and bicarbonate-related absorptions. Bayesian inference applied to integrated FTIR band areas provides statistically robust evidence that sustained accumulation of formate-like species is intrinsically associated with irradiation of the hybrid material. This conclusion is independently corroborated by self-organizing map analysis of the full spectral fingerprints and cannot be explained by simple physical mixing of the parent oxides or background carbonate adsorption. Although the FTIR and statistical analyses are consistent with surface-bound formate formation, definitive molecular identification beyond vibrational and statistical evidence is outside the scope of the present work.

Total organic carbon remains below the detection limit throughout the experiments, while inorganic carbon decreases significantly between 24 and 48 h of irradiation, a trend supported by both classical statistical analysis and Bayesian inference. Taken together, these observations indicate that carbon species generated under irradiation do not accumulate in the bulk aqueous phase but are instead preferentially stabilized at the solid–liquid interface. The delayed yet sustained emergence of formate-related vibrational features is therefore consistent with a progressive transformation of surface-associated inorganic carbon species into more persistent, surface-stabilized carboxylates, with formate representing a plausible dominant surface species. Thermal regeneration experiments further reveal partial reversibility of the irradiation-induced spectral features, indicating that while a fraction of the surface-associated species can be removed under mild thermal treatment, strong Bayesian evidence indicates that a residual contribution remains stabilized at the surface.

Overall, the CeO_2_–SnO_2_ hybrid serves as a useful model system for probing photoassisted bicarbonate conversion at oxide surfaces under aqueous conditions. To the best of our knowledge, this study provides the first evidence that irradiation can induce the formation of surface-bound, formate-like species from aqueous bicarbonate in CeO_2_–SnO_2_ hybrids. This distinguishes the present work from previous studies, which have largely focused on the electrocatalytic reduction of bicarbonate or CO_2_ over Sn-based materials to yield formate or formic acid. It also differs fundamentally from earlier reports on the electrocatalytic reduction of gaseous CO_2_ to formic acid using SnO_2_ supported on CeO_2_, as those systems did not involve a truly integrated CeO_2_–SnO_2_ hybrid nor a photoassisted pathway in aqueous bicarbonate media.

More broadly, this study highlights the value of combining surface-sensitive spectroscopic techniques with probabilistic statistical frameworks to resolve subtle, time-dependent surface transformations and to distinguish irradiation-driven interfacial chemistry from experimental variability in heterogeneous surface conversion systems. Future work of interest includes evaluating the system under natural or simulated solar irradiation and pursuing complementary spectroscopic approaches to further elucidate the identity, stability, and dynamics of the surface-associated carbon species formed under these conditions.

## Supplementary Material



## Data Availability

The processed data supporting the findings of this study are available within the manuscript and Supporting Information. Raw data are available from the corresponding author upon reasonable request.

## Notes

Detailed ATR-FTIR data processing procedures, band integration methodology, and overlap-correction strategy; description of neural network architecture and training for spectral feature selection; full Bayesian inference frameworks for FTIR band areas, inorganic carbon (IC), and thermal regeneration analyses, including priors, likelihoods, and sampling parameters; convergence diagnostics and posterior summaries; self-organizing map (SOM) analysis methodology and clustering procedures; reproducibility details and software implementation; additional IC and total organic carbon (TOC) data sets; robustness analysis of Bayesian models under alternative specifications; supplementary figures and tables supporting irradiation, control, and thermal regeneration experiments (PDF)
