# Supplementary material for: Light-Assisted Conversion of Aqueous CO2 (Bicarbonate) into Surface Reduced Species by a CeO2–SnO2 Hybrid
Source: ACS Omega. 2026 Apr 27;11(18):27034–49. doi: 10.1021/acsomega.6c00620 (PMC13177208; doi:10.1021/acsomega.6c00620)
Supplement: Supplementary file 1 [file ao6c00620_si_001.pdf]

Supporting Information for "Light-Assisted Conversion of Aqueous CO_2 (Bicarbonate) into Surface Reduced Species by a CeO_2–SnO_2 Hybrid"

Lucas Hansen[a]*, Ubiratan Hack[b], Marco Antônio Rodrigues Siqueira[b], Daniel Eduardo Weibel [a]*

[a]Instituto de Química, Universidade Federal do Rio Grande do Sul, Avenida Bento Gonçalves 9500 Porto Alegre RS Brazil 91501-970

[b]Laboratório Aquário, Universidade Feevale, RS 239 2755 Novo Hamburgo RS Brazil 93525-075

*Corresponding authors. E-mails: 00191288@ufrgs.br; danielw@iq.ufrgs.br

ATR-FTIR data processing and band integration

ATR-FTIR spectra were collected in absorbance mode and interpolated onto a common wavenumber grid prior to further analysis. Baseline correction was performed using a local polynomial baseline fitted outside literature-assigned vibrational regions associated with bicarbonate, carboxylate, and C–H modes. Baseline-corrected spectra were subsequently used for all quantitative analyses. This approach was adopted to remove slowly varying background contributions arising from instrumental drift, scattering, and broad absorption features, while preserving chemically meaningful vibrational information. Fitting the baseline only outside literature-assigned vibrational regions minimizes distortion of true absorption bands and prevents artificial suppression or enhancement of integrated intensities, thereby ensuring that subsequent numerical integration reflects genuine chemical contributions rather than baseline artifacts.

Integrated band intensities were obtained by direct numerical integration of absorbance over predefined wavenumber intervals corresponding to literature-reported vibrational assignments for adsorbed bicarbonate and formate species. To avoid double counting in spectral regions where bicarbonate- and formate-associated bands partially overlap, union masks were constructed for each chemical family. Absorbance contributions belonging to the opposing family were explicitly excluded prior to integration, ensuring that the resulting band areas were mutually exclusive while remaining fully consistent with established vibrational assignments.

The neural-network output was not used to define new vibrational bands, alter spectral boundaries, or participate directly in quantification. Instead, it served exclusively as a relevance-based filter that identified chemically meaningful, literature-assigned bands and suppressed noise-dominated spectral regions. As a result, band integration remained anchored to prior spectroscopic knowledge while benefiting from objective band selection derived from the experimental dataset itself.

Neural-network architecture and training

A shallow autoencoder neural network was employed as an unsupervised feature-selection tool for the FTIR dataset. Input spectra were preprocessed using standard normal variate (SNV) normalization followed by Savitzky–Golay first-derivative filtering to minimize baseline variability and emphasize subtle, overlapping vibrational features. The autoencoder consisted of a symmetric encoder–latent–decoder architecture with fully connected layers, comprising a small number of hidden layers and a low-dimensional latent bottleneck. Specifically, the network included an input layer matching the dimensionality of the preprocessed spectra, one hidden encoding layer with a reduced number of neurons, a compact latent layer, and a mirrored decoding layer, resulting in a total depth of three trainable layers between input and output.

In this context, the term "shallow" denotes the intentionally limited network depth and parameter count, chosen to reduce model complexity, avoid overfitting, and preserve interpretability under the small-sample conditions typical of spectroscopic datasets. The network was trained to minimize reconstruction error using the full preprocessed spectral dataset without the use of predefined classes or response variables. Training was performed with fixed random seeds to ensure reproducibility. Neuron relevance weights derived from the trained autoencoder were used to identify spectral regions that contributed most strongly to reconstruction fidelity across the dataset. These relevance profiles were subsequently intersected with literature-assigned vibrational ranges to guide objective band selection.

Importantly, the neural network was not used to infer chemical identities or to generate new spectral features. Its role was limited to data-driven relevance weighting within

predefined chemical constraints, thereby avoiding black-box interpretation and preserving physical interpretability.

Bayesian analysis of FTIR band areas

Bayesian inference was applied to NN-selected, overlap-corrected FTIR band areas to quantify irradiation-dependent differences in surface-bound carbon species while explicitly accounting for experimental uncertainty. Integrated band areas were strictly positive and exhibited right-skewed distributions; therefore, all values were log-transformed and standardized prior to modeling. On the transformed scale, band areas for each experimental condition were modeled independently using a Student's t likelihood with fixed degrees of freedom ($\nu = 7$), providing robustness to residual variability and potential outliers common in spectroscopic datasets. Each condition-specific mean parameter was assigned a weakly informative normal prior, while a shared residual scale parameter was assigned a weakly informative half-normal prior.

Weakly informative priors were selected to regularize parameter estimation while remaining agnostic with respect to the expected magnitude of the effects. The sample-specific mean parameters were assigned standard normal priors, $\mu_i \sim N(0, 1)$, which are centered at zero on the log-transformed, standardized scale of the data and therefore encode no directional preference while constraining the parameters to physically reasonable ranges relative to the observed variability. This choice prevents implausibly large excursions of μ_i in small-sample settings without imposing substantive prior information.

The shared residual scale parameter was assigned a half-normal prior, $\sigma \sim \mathrm{HalfNormal}(1)$, which enforces the natural non-negativity constraint on variance parameters while remaining weakly informative on the standardized scale. This prior places most probability mass on modest residual variability but retains sufficiently heavy tails to accommodate larger dispersion if supported by the data. Together, these priors provide numerical stability and guard against overfitting while allowing the posterior to be driven primarily by the experimental observations.

Posterior inference was performed using Hamiltonian Monte Carlo with the No-U-Turn Sampler (NUTS), employing four independent chains with 3,000 warm-up iterations followed by 6,000 sampling iterations per chain and a target acceptance probability of 0.99. Posterior samples were used to compute condition-specific posterior distributions and pairwise posterior probabilities of the form $P(\mu_i > \mu_{\square})$, enabling direct probabilistic comparison of surface-bound species across irradiation times without reliance on dichotomous significance thresholds.

Bayesian analysis of inorganic carbon (IC) measurements

In parallel with classical hypothesis testing, inorganic carbon (IC) measurements were analyzed using a Bayesian probabilistic framework to provide complementary inference under small-sample conditions. IC concentrations corresponding to each irradiation time were modeled using a non-hierarchical Student's t likelihood with fixed degrees of freedom ($\nu = 7$), accommodating potential deviations from normality and increased uncertainty associated with limited replication. Group-specific mean IC values were assigned weakly informative normal priors, while a shared residual scale parameter was assigned a weakly informative half-normal prior. Posterior inference was obtained using Hamiltonian Monte Carlo NUTS sampling with four independent chains, each comprising 3,000 warm-up iterations followed by 6,000 sampling iterations and a target acceptance probability of 0.99. The resulting posterior distributions enabled direct probabilistic assessment of IC differences between irradiation times, including posterior probabilities for relative decreases in IC, thereby reinforcing trends identified by classical tests while avoiding reliance on asymptotic assumptions.

Bayesian analysis of thermal regeneration data

Bayesian inference was additionally applied to FTIR band areas obtained after thermal regeneration to assess probabilistic changes in surface-bound carbon species following thermal treatment. Regenerated-sample band areas were analyzed using the same non-hierarchical Bayesian framework employed for the irradiation-dependent FTIR data,

including identical preprocessing, likelihood specification, priors, and sampling parameters, ensuring methodological consistency across analyses. As in the irradiation analysis, band areas were log-transformed and standardized, and a Student's t likelihood with fixed degrees of freedom ($\nu = 7$) was used to model residual variability. Posterior samples were propagated to compute condition-specific posterior distributions and pairwise posterior probabilities comparing regenerated, irradiated, and control conditions. This unified Bayesian treatment enabled direct probabilistic evaluation of irradiation-induced surface accumulation and its thermal reversibility on a common inferential basis, while explicitly accounting for experimental uncertainty and avoiding additional model assumptions.

Across all Bayesian analyses, a Student's t likelihood was employed instead of a Gaussian likelihood to provide robustness against residual non-normality, mild outliers, and heterogeneity arising from spectral preprocessing, numerical integration, and small sample sizes. Integrated FTIR band areas and inorganic carbon measurements are continuous, positive quantities that can exhibit right-skewness and occasional deviations from ideal normal behavior, particularly under limited replication. The degrees of freedom of the Student's t distribution were fixed at $\nu = 7$, representing a conservative compromise between robustness and numerical stability. This choice yields heavier tails than a normal distribution, reducing sensitivity to individual observations, while avoiding the excessive variance inflation and unstable inference associated with very low degrees of freedom. Fixing ν rather than estimating it from the data further avoids overparameterization and improves sampling efficiency under small-sample conditions. The same likelihood specification was applied consistently across all Bayesian analyses to maintain internal coherence and comparability of posterior inferences.

Convergence metrics of Bayesian analyses

Table S1 shows the convergence diagnostics for the Bayesian analysis of preprocessed ATR-FTIR spectra with respect to formate-related bands, while Table S2 shows the same diagnostics for bicarbonate-related bands:

Table S1. Posterior summary statistics for the group-level mean parameters (μ) obtained from the Bayesian analysis of preprocessed ATR-FTIR spectra for formate-related bands of irradiation and control experiments. The parameters correspond, respectively, to the following experimental conditions: μ[0], 48 h irradiation; μ[1], 24 h irradiation; μ[2], 6 h irradiation; μ[3], 48 h dark control; μ[4], bare hybrid material; μ[5], physical mixture after 48 h irradiation; and μ[6], bare physical mixture. Reported values include posterior means, standard deviations (SD), 95 % highest density intervals (HDI), Monte Carlo standard errors (MCSE), effective sample sizes (ESS), and potential scale reduction factors ($\hat{R}$). All μ and σ values are reported on the log-transformed, standardized scale used for model fitting; values therefore reflect relative differences in overlap-corrected FTIR band areas rather than absolute intensities. The parameter σ represents the shared residual scale of the model.

Parameter	Mean	SD	HDI 2.5%	HDI 97.5%	MCSE mean	MCSE sd	ESS bulk	ESS tail	$\hat{R}$
**μ[0]**	1.187	0.583	-0.140	2.142	0.00822	0.00725	6029	10148	1.001
**μ[1]**	0.883	0.533	-0.331	1.840	0.00628	0.00743	9316	9957	1.001
**μ[2]**	0.090	0.474	-0.903	1.143	0.00345	0.00669	19674	11729	1.002
**μ[3]**	-0.152	0.467	-1.158	0.857	0.00335	0.00711	21098	10731	1.003
**μ[4]**	-0.240	0.478	-1.221	0.836	0.00366	0.00715	18464	10076	1.002
**μ[5]**	-0.494	0.484	-1.458	0.593	0.00433	0.00648	15847	10940	1.002
**μ[6]**	-1.288	0.598	-2.176	0.113	0.00899	0.00749	5156	9974	1.001
**sigma**	0.488	0.355	0.018	1.166	0.00899	0.00311	981	763	1.005

Table S2. Posterior summary statistics for the group-level mean parameters (μ) obtained from the Bayesian analysis of preprocessed ATR-FTIR spectra for bicarbonate-related bands of irradiation and control experiments. The parameters correspond, respectively, to the following experimental conditions: μ[0], 48 h irradiation; μ[1], 24 h irradiation; μ[2], 6 h irradiation; μ[3], 48 h dark control; μ[4], bare hybrid material; μ[5], physical mixture after 48 h irradiation; and μ[6], bare physical mixture. Reported values include posterior means, standard deviations (SD), 95 % highest density intervals (HDI), Monte Carlo standard errors (MCSE), effective sample sizes (ESS), and potential scale reduction factors ($\hat{R}$). All μ and σ values are reported on the log-transformed, standardized scale used for model fitting; values therefore reflect relative differences in overlap-corrected FTIR band areas rather than absolute intensities. The parameter σ represents the shared residual scale of the model.

Parameter	Mean	SD	HDI 2.5%	HDI 97.5%	MCSE mean	MCSE sd	ESS bulk	ESS tail	$\hat{R}$
**μ[0]**	-0.169	0.471	-1.150	0.863	0.00357	0.00678	18489	10481	1.002
**μ[1]**	-1.038	0.559	-1.948	0.269	0.00701	0.00696	7727	10372	1.000
**μ[2]**	-0.574	0.506	-1.527	0.599	0.00476	0.00680	13295	10596	1.001
**μ[3]**	0.292	0.474	-0.763	1.271	0.00387	0.00662	17037	10677	1.002

**μ[4]**	-0.322	0.483	-1.288	0.785	0.00398	0.00691	16341	10064	1.000
**μ[5]**	1.544	0.675	-0.008	2.483	0.0106	0.00697	4427	10774	1.000
**μ[6]**	0.236	0.481	-0.867	1.192	0.00373	0.00705	18191	10570	1.001
**sigma**	0.493	0.356	0.014	1.165	0.00818	0.00317	1154	764	1.002

All model parameters in Tables S1 and S2 exhibit excellent convergence and sampling performance. Potential scale reduction factors ($\hat{R}$) are close to unity for all parameters ($\hat{R}$ ≤ 1.002), indicating that the independent Markov chains converged to the same posterior distribution and that no between-chain inconsistencies are present. No divergent transitions were observed during sampling. Effective sample sizes (ESS) for the group-level mean parameters μ are high, exceeding 4,000 for all conditions and reaching values close to 20,000 for several parameters, demonstrating efficient exploration of the posterior space and minimal autocorrelation in the sampled chains. Monte Carlo standard errors (MCSE) for both posterior means and standard deviations are small relative to the corresponding posterior uncertainties, confirming that numerical sampling error is negligible compared to the intrinsic variability of the data. This indicates that the reported posterior summaries are stable and not sensitive to stochastic sampling effects. The shared residual scale parameter (σ) also shows acceptable convergence, with $\hat{R}$ close to unity and adequate effective sample sizes, supporting the reliability of uncertainty estimates across conditions. Collectively, these diagnostics confirm that posterior inferences are robust, well-resolved, and not limited by insufficient sampling or convergence issues. The observed uncertainty in the posterior distributions therefore reflects genuine experimental variability rather than artifacts of the inference procedure.

Convergence diagnostics for the Bayesian analysis of the inorganic carbon (IC) data are presented in table S3:

Table S3. Posterior summary statistics for the group-level mean parameters (μ) obtained from the Bayesian analysis of the inorganic carbon data. The parameters correspond, respectively, to the following experimental conditions: μ[0], 48 h irradiation; μ[1], 24 h irradiation. Reported values include posterior means, standard deviations (SD), 95 % highest density intervals (HDI), Monte Carlo standard errors (MCSE), effective sample sizes (ESS), and potential scale reduction factors ($\hat{R}$). The parameter σ represents the shared residual scale of the model.

Parameter	Mean	SD	HDI 2.5%	HDI 97.5%	MCSE mean	MCSE sd	ESS bulk	ESS tail	R̂
**μ[0]**	1072.910	39.804	994.621	1146.257	0.388	0.695	1226	8918	1.001
**μ[1]**	964.070	43.035	880.804	1043.446	0.378	0.631	14463	978	1.000
**sigma**	62.871	33.469	20.733	121.535	0.396	0.646	8269	9228	1.001

All parameters in Table S3 exhibit excellent convergence and sampling performance. Potential scale reduction factors (R̂) are effectively unity for all parameters (R̂ ≤ 1.001), indicating consistent convergence of the independent Markov chains to the same posterior distribution. No divergent transitions were observed during sampling. Effective sample sizes are high for all parameters, exceeding 8,000 for both the group-level mean parameters and the residual scale parameter, demonstrating efficient posterior exploration and low autocorrelation within chains. Monte Carlo standard errors for both posterior means and standard deviations are small relative to the corresponding posterior uncertainties, confirming that numerical sampling error is negligible. These diagnostics indicate stable, well-mixed chains and reliable posterior summaries, with no evidence of convergence or sampling pathologies.

Finally, Tables S4 and S5 present convergence diagnostics for the preprocessed ATR-FTIR spectra for formate- and bicarbonate-related bands, respectively, of the thermally regenerated samples of the hybrid material as well as of the air-exposed sample of the material:

Table S4. Posterior summary statistics for the group-level mean parameters (μ) obtained from the Bayesian analysis of preprocessed ATR-FTIR spectra of regeneration experiments for formate-related bands. The parameters correspond, respectively, to the following experimental conditions: μ[0], 4 h regeneration at 200°C; μ[1], 2 h regeneration at 200°C; μ[2], air-exposed hybrid material. Reported values include posterior means, standard deviations (SD), 95 % highest density intervals (HDI), Monte Carlo standard errors (MCSE), effective sample sizes (ESS), and potential scale reduction factors (R̂). All μ and σ values are reported on the log-transformed, standardized scale used for model fitting; values therefore reflect relative differences in overlap-corrected FTIR band areas rather than absolute intensities. The parameter σ represents the shared residual scale of the model.

Parameter	Mean	SD	HDI 2.5%	HDI 97.5%	MCSE mean	MCSE sd	ESS bulk	ESS tail	R̂

**μ[0]**	0.457	0.545	-0.788	1.509	0.00568	0.00670	9925	9588	1.001
**μ[1]**	0.556	0.562	-0.765	1.572	0.00644	0.00723	8774	9065	1.001
**μ[2]**	-1.002	0.641	-2.030	0.490	0.00883	0.00763	5899	9085	1.001
**sigma**	0.610	0.466	0.009	1.535	0.00868	0.00390	1774	1174	1.004

Table S5. Posterior summary statistics for the group-level mean parameters (μ) obtained from the Bayesian analysis of preprocessed ATR-FTIR spectra of regeneration experiments for bicarbonate-related bands. The parameters correspond, respectively, to the following experimental conditions: μ[0], 4 h regeneration at 200°C; μ[1], 2 h regeneration at 200°C; μ[2], air-exposed hybrid material. Reported values include posterior means, standard deviations (SD), 95 % highest density intervals (HDI), Monte Carlo standard errors (MCSE), effective sample sizes (ESS), and potential scale reduction factors ($\hat{R}$). All μ and σ values are reported on the log-transformed, standardized scale used for model fitting; values therefore reflect relative differences in overlap-corrected FTIR band areas rather than absolute intensities. The parameter σ represents the shared residual scale of the model.

**Parameter**	**Mean**	**SD**	**HDI 2.5%**	**HDI 97.5%**	**MCSE mean**	**MCSE sd**	**ESS bulk**	**ESS tail**	**$\hat{R}$**
**μ[0]**	-0.909	0.628	-1.919	0.581	0.00863	0.00777	6129	8429	1.001
**μ[1]**	0.088	0.538	-1.107	1.188	0.00506	0.00732	11607	9549	1.001
**μ[2]**	0.827	0.604	-0.562	1.871	0.00762	0.00717	7372	8920	1.001
**sigma**	0.616	0.461	0.013	1.496	0.00845	0.00367	1867	1268	1.002

All parameters in Tables S4 and S5 exhibit excellent convergence and sampling performance. Potential scale reduction factors ($\hat{R}$) are close to unity for all parameters ($\hat{R} \leq 1.002$), indicating consistent convergence of the independent Markov chains to the same posterior distributions. Effective sample sizes are high for all group-level mean parameters, with bulk ESS values generally exceeding 5,000 and tail ESS values remaining well above commonly accepted thresholds, demonstrating efficient posterior exploration and low autocorrelation within chains. Monte Carlo standard errors for both posterior means and standard deviations are small relative to the corresponding posterior uncertainties, confirming that numerical sampling error is negligible. The shared residual scale parameters (σ) in both models also show acceptable convergence behavior, with $\hat{R}$ values near unity and adequate effective sample sizes given the limited size of the regeneration datasets. These diagnostics again indicate stable, well-mixed chains and reliable posterior summaries, with no evidence of convergence or sampling pathologies in either the formate- or bicarbonate-band analyses.

Self-organizing map (SOM) analysis

Self-organizing map (SOM) analysis was employed as an independent, unsupervised multivariate approach to validate and contextualize the trends identified by Bayesian inference, without imposing any a priori assumptions regarding sample grouping or chemical identity. Preprocessed ATR-FTIR spectra, subjected to standard normal variate (SNV) normalization and first-derivative filtering to minimize baseline effects and emphasize subtle band-shape variations, were projected onto a two-dimensional SOM lattice using the *MiniSom* Python implementation. The network was trained on the full spectral window using a competitive learning algorithm, ensuring preservation of topological relationships such that spectra with higher similarity were mapped to neighboring neurons in the latent space.

Following training, clustering was performed on the SOM neuron weight vectors using k-means analysis, with the optimal number of clusters determined automatically via an internal cluster validity criterion, thereby avoiding subjective selection of cluster count. Importantly, the clustering was applied to the learned spectral prototypes rather than directly to the raw spectra, allowing noise-reduced, topology-aware classification that reflects intrinsic spectral structure rather than experimental variance.

To probe the chemical origin of the observed spectral differentiation, a formate-specific component plane was constructed by averaging neuron weight intensities over vibrational regions commonly assigned to formate-related modes in the literature, while explicitly excluding spectral intervals dominated by water absorption or bicarbonate features. This component plane does not represent a quantitative concentration map but instead highlights relative enrichment or depletion of formate-associated spectral contributions across the SOM lattice.

Projection of individual samples onto the trained SOM revealed a systematic spatial organization consistent with irradiation time, with spectra obtained after prolonged irradiation preferentially occupying neurons associated with higher values in the formate-related component plane. In contrast, control experiments, including dark conditions and physical mixtures of the parent oxides, were distributed in regions characterized by low

or negligible formate-associated signal, indicating that the observed SOM separation cannot be attributed to generic spectral variability or preprocessing artifacts.

Taken together, the SOM analysis provides an orthogonal, fully unsupervised confirmation of the Bayesian results, demonstrating that irradiation induces reproducible, time-dependent spectral reorganization aligned with formate-related vibrational features. The convergence of probabilistic inference and topology-preserving machine-learning analysis strengthens the conclusion that the observed spectral evolution is intrinsic to the CeO_2–SnO_2 hybrid interface rather than a consequence of random noise, baseline effects, or inherited surface properties from the parent oxides.

Reproducibility and software

All data processing, statistical analysis, and visualization were performed using the Python scientific computing ecosystem. Key packages included *NumPy*, *SciPy*, *pandas*, *matplotlib*, *scikit-learn*, *MiniSom*, *PyMC*, and *ArviZ.* Random seeds were fixed where applicable to ensure reproducibility of neural-network training and Bayesian sampling. The scripts used for data analysis are available from the corresponding author upon reasonable request.

Inorganic and total organic carbon results

Table S6 summarizes the inorganic carbon (IC) and total organic carbon (TOC) concentrations measured in aqueous suspensions of the CeO_2–SnO_2 hybrid material after 24 h and 48 h of visible-light irradiation in 0.1 M $NaHCO_3$. Measurements were performed on independent replicate experiments to assess reproducibility. TOC concentrations were below the instrumental detection limit in all cases, indicating the absence of detectable dissolved organic carbon in solution following irradiation.

The IC measurements show a consistent decrease in inorganic carbon concentration between 24 h and 48 h of irradiation, despite the absence of measurable TOC. This behavior suggests that irradiation induces a net transformation or redistribution of

inorganic carbon species that does not result in the accumulation of dissolved organic products in the aqueous phase. The lack of detectable TOC is consistent with preferential stabilization of reduced carbon species at the solid–liquid interface, adsorption of organic intermediates onto the hybrid material surface, or conversion pathways that do not yield soluble organic carbon under the experimental conditions. These observations support the interpretation that carbon conversion under irradiation is predominantly surface-associated rather than occurring in the bulk solution.

Table S6. Inorganic carbon (IC) and total organic carbon (TOC) concentrations measured in aqueous suspensions of the CeO_2–SnO_2 hybrid material after 24 h and 48 h of visible-light irradiation in 0.1 M $NaHCO_3$. Values correspond to independent replicate experiments. TOC values were below the detection limit in all cases, indicating the absence of detectable dissolved organic carbon in solution despite irradiation.

Sample	Conditions	Inorganic carbon (mg/L)	Total organic carbon (mg/L)
**1**	24 h irradiation, hybrid material suspended in 0.1 M $NaHCO_3$	1040.0	0
**2**	24 h irradiation, hybrid material suspended in 0.1 M $NaHCO_3$	1090.0	0
**3**	24 h irradiation, hybrid material suspended in 0.1 M $NaHCO_3$	1090.0	0
**4**	48 h irradiation, hybrid material suspended in 0.1 M $NaHCO_3$	899.3	0
**5**	48 h irradiation, hybrid material suspended in 0.1 M $NaHCO_3$	969.9	0
**6**	48 h irradiation, hybrid material suspended in 0.1 M $NaHCO_3$	1020.0	0

Bayesian inference results for bicarbonate-related FTIR bands across irradiation and control experiments

Figure S1 summarizes the results of Bayesian pairwise comparisons of the overlap-corrected integrated areas associated with bicarbonate-related vibrational features. The analysis reveals a consistently higher posterior probability for the air-exposed hybrid material (Bare HM) to exhibit larger bicarbonate-related surface contributions compared to the irradiated samples, particularly the 24 h and 48 h irradiation conditions. In contrast, prolonged irradiation does not increase the probability of enhanced bicarbonate-associated band intensity relative to the non-irradiated material, except for the hybrid material sample irradiated for 48h compared to the air-exposed hybrid. These results indicate that irradiation does not overall promote bicarbonate accumulation at the hybrid surface. Instead, the Bayesian analysis supports the interpretation that bicarbonate-related surface features are diminished upon irradiation, consistent with a redistribution of surface carbon species toward alternative, irradiation-induced forms rather than increased bicarbonate adsorption.

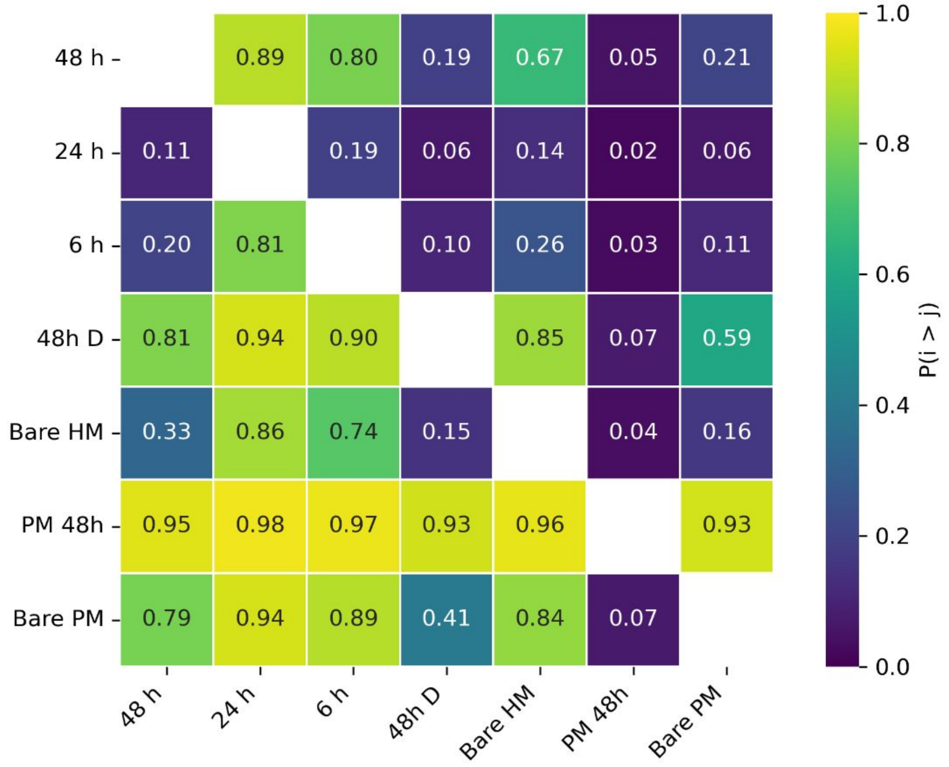

Figure S1. Heatmap showing pairwise Bayesian posterior probabilities $P(i > j)$for overlap-corrected integrated areas of bicarbonate-related FTIR bands across the different samples and irradiation conditions. Each cell represents the posterior probability that the bicarbonate-associated band area of sample i (row) exceeds that of sample j (column). Values close to 1 indicate strong evidence that sample i contains a larger bicarbonate-related surface contribution than sample j, whereas values close to 0 indicate the opposite. Diagonal elements are omitted for clarity.

Bayesian inference results of formate- and bicarbonate-related ATR-FTIR bands in thermally regenerated hybrid material

Figure S2 shows the Bayesian probability-of-superiority (PS) matrices computed for the integrated ATR-FTIR band areas associated with formate- and bicarbonate-related vibrational regions in the thermally regenerated hybrid material.

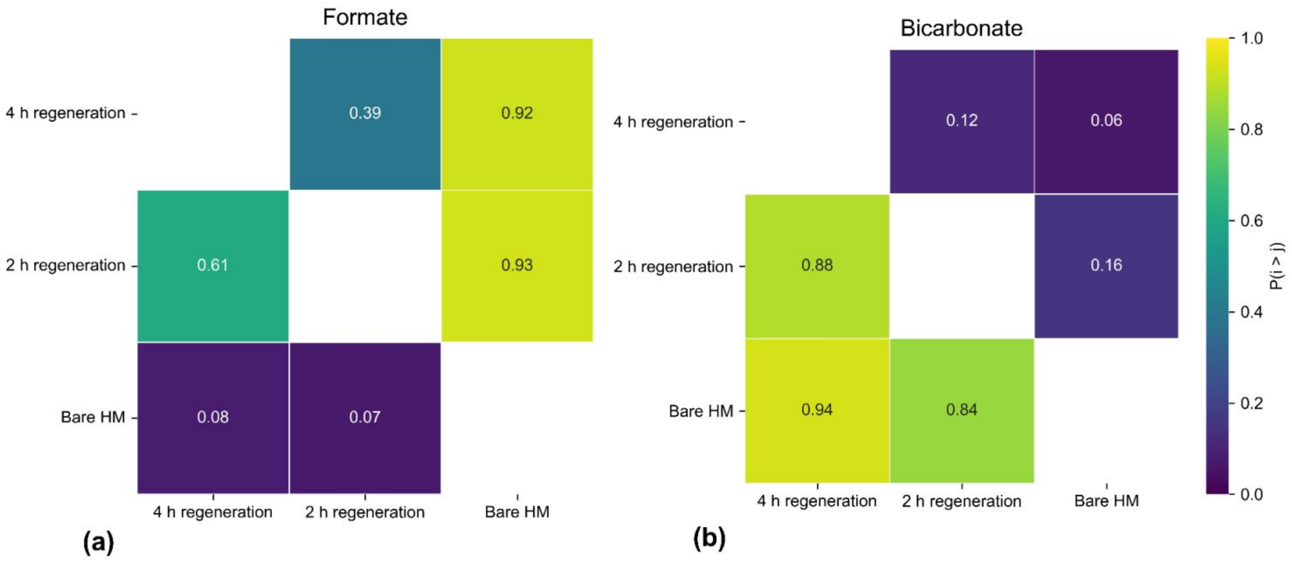

Figure S2. Bayesian pairwise posterior probabilities for overlap-corrected FTIR band areas associated with (a) formate and (b) bicarbonate after thermal regeneration (2 h and 4 h at 200 °C), shown relative to the physical mixture reference (Bare PM). Each cell reports the posterior probability P(i > j) that the band intensity of sample *i* exceeds that of sample *j*.

The resulting PS heatmaps are shown in Figure S3 for (a) formate-related bands and (b) bicarbonate-related bands. Each cell represents the posterior probability that the integrated band area of the condition listed on the y-axis exceeds that of the condition listed on the x-axis, $P(A_i > A_j)$. Values approaching 1 indicate strong support that the row condition exhibits larger band areas than the column condition, whereas values approaching 0 indicate the opposite relationship. Diagonal elements are omitted because self-comparisons are not defined.

Total FTIR areas

For completeness, the total integrated absorbance areas of the ATR-FTIR spectra were calculated for each sample. The integrated area was determined numerically using the trapezoidal rule over the analyzed spectral region (650–3050 cm^{-1}). In addition to the overall spectral area, integrated areas associated with the formate-related and bicarbonate-related vibrational regions were obtained by summing the band integrals within the literature-assigned windows used in the main analysis.

The resulting values therefore represent the total spectral absorbance within the analyzed FTIR window, as well as the integrated contributions of the formate-related and bicarbonate-related vibrational bands. Together, these quantities provide a quantitative measure of the relative spectral contributions of the different surface species present on the catalyst surface. Table S7 shows the total integrated areas for each experimental condition

Table S7. Total integrated ATR-FTIR absorbance areas calculated for the analyzed spectral region (650–3050 cm^{-1}), together with the integrated contributions of the formate-related and bicarbonate-related vibrational bands used in the main analysis. Band areas were obtained by numerical integration using the trapezoidal rule within the literature-assigned vibrational windows. The resulting values provide a quantitative measure of the relative spectral contributions of formate-like and bicarbonate species on the catalyst surface after irradiation and in the control samples.

Sample	Conditions	Integrated total area, a.u.
Bare PM	Air-exposed physical mixture of parent oxides	0.86
Bare HM	Air-exposed hybrid material	0.74
48h D	48 h dark experiment, hybrid material suspended in 0.1 M $NaHCO_3$	0.12
6 h	6 h irradiation, hybrid material suspended in 0.1 M $NaHCO_3$	0.17
24 h	24 h irradiation, hybrid material suspended in 0.1 M $NaHCO_3$	26.89

48 h	48 h irradiation, hybrid material suspended in 0.1 M $NaHCO_3$	50.40
PM 48 h	48 h irradiation, physical mixture of parent oxides suspended in 0.1 M $NaHCO_3$	0.12

For the 6 h and 48 h dark samples, the integrated formate-band areas exceed the total spectral area obtained from the global integration window. This occurs because the band integrations employ local baseline subtraction within each vibrational window, whereas the total spectral area is computed using a single global baseline across the entire spectrum. In spectra exhibiting broad background contributions (e.g., from adsorbed water or surface hydroxyl groups), the local baseline within narrow bands may lie below the global baseline, leading to slightly larger integrated band areas. This artifact does not affect the qualitative or quantitative trends observed across the irradiation series.

Robustness assessment of Bayesian analysis of FTIR data

To assess the sensitivity of the Bayesian analysis of FTIR data to modeling assumptions, robustness checks were performed by systematically varying the Student-t likelihood degrees of freedom (ν = 4, 7, 15), the prior scale of the dispersion parameter (σp = 0.5, 1.0, 2.0), and the variance structure (shared versus group-specific σ). For each combination, posterior predictive checks were generated for the overlap-corrected integrated areas associated with the formate- and bicarbonate-related spectral regions, yielding 18 model variants for each spectral family. The purpose of these tests was not to identify an alternative preferred model, but to determine whether the qualitative conclusions of the spectral analysis remained stable across a broad range of reasonable statistical specifications. Because the dataset consists of a small number of condition-level observations rather than multiple independent replicates per condition, the baseline model uses a non-hierarchical structure; the robustness sweep therefore focuses on sensitivity to likelihood tail behavior, dispersion priors, and variance parameterization rather than hierarchical pooling.

For the formate-related bands, the posterior predictive envelopes (Figure S3) showed highly consistent behavior across all model variants. The predicted mean trend and the relative ordering of experimental conditions remained unchanged despite variations in ν, prior scale, or variance structure. Increasing the dispersion prior or allowing group-specific variances broadened the predictive intervals, as expected, but did not alter the predicted pattern of decreasing standardized log-area values from irradiated hybrid samples toward the control materials. This stability indicates that the inference regarding the progressive formation of formate-like species on the hybrid surface with irradiation time is not dependent on specific modeling assumptions.

A similar robustness pattern was observed for the bicarbonate-related spectral integrations (Figure S4). Across all tested specifications, posterior predictive checks reproduced the same qualitative structure of the observations, with only the width of the predictive intervals varying according to the assumed dispersion prior or variance structure. Models with group-specific variance parameters produced wider uncertainty bands, reflecting the additional flexibility of the variance structure in a dataset with few observations, but the predicted trend across experimental conditions remained unchanged. These results demonstrate that the statistical interpretation of the bicarbonate-associated spectral features is likewise stable across a wide range of plausible modeling choices.

Overall, the robustness analysis shows that the statistical interpretation of the ATR-FTIR spectral integrations is stable across a broad range of modeling assumptions. Variations in likelihood tail behavior, prior scale for dispersion, and variance structure mainly affected the width of the posterior predictive intervals but did not alter the predicted ordering or qualitative trends of the experimental conditions. Consequently, the conclusions drawn from the neural-network-assisted band selection and overlap-corrected integration procedure remain robust under multiple alternative probabilistic models.

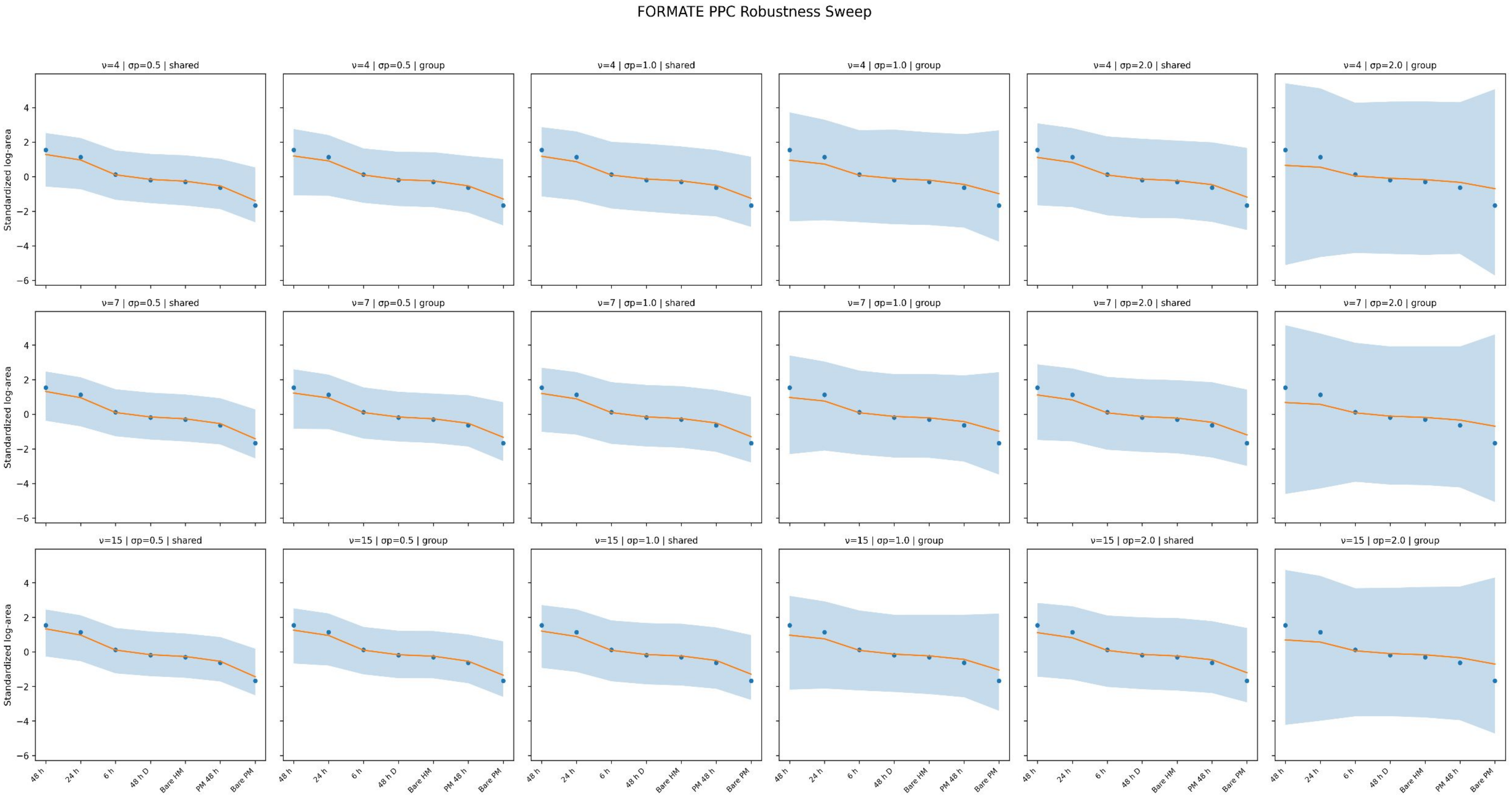

Figure S3. Posterior predictive checks for the Bayesian FTIR analysis of formate-related bands under alternative model specifications (ν, σ prior scale, and variance structure); blue points show observed standardized log-integrated areas, the orange line the predictive mean, and the blue shaded envelope the posterior predictive interval.

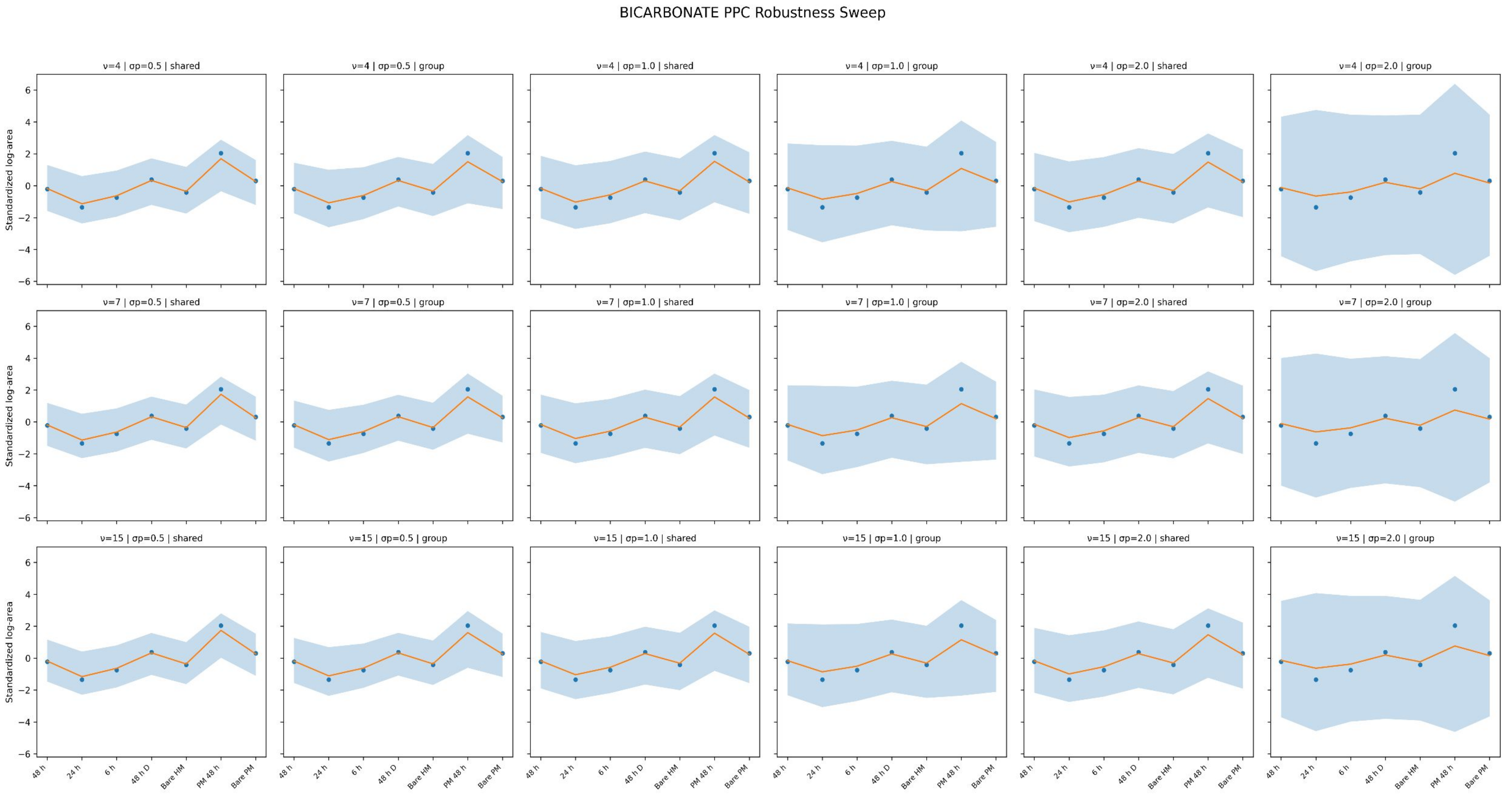

Figure S4. Posterior predictive checks for the Bayesian FTIR analysis of bicarbonate-related bands under alternative model specifications (ν, σ prior scale, and variance structure); blue points show observed standardized log-integrated areas, the orange line the predictive mean, and the blue shaded envelope the posterior predictive interval.

Convergence diagnostics for the robustness models indicated generally stable sampling behavior across the tested configurations. For the formate-related band analysis, all model variants exhibited good convergence, with $\hat{R}$ values below 1.01 and effective sample sizes exceeding typical reliability thresholds. For the bicarbonate-related bands, most model configurations also showed satisfactory convergence; however, two extreme heavy-tailed variants ($\nu = 4$ with a shared variance parameter) displayed reduced chain mixing, reflected in slightly elevated $\hat{R}$ values and lower effective sample sizes. All other configurations converged well according to standard diagnostics, indicating stable posterior sampling for the majority of tested model specifications.
